# Complexes of Zinc(II) Chloride with *N*-Vinyl-, *N*-Allyl- and *N*-Propargylimidazoles: Structural, Theoretical and Biological Studies

**DOI:** 10.3390/ph19060874

**Published:** 2026-05-31

**Authors:** Vladimir S. Tyurin, Victoria S. Babasieva, Mikhail S. Grigoriev, Lidiya N. Parshina, Ilya A. Zamilatskov, Elena A. Smolyarchuk, Olga V. Nesterova, Vladislav N. Turenko, Tatiana I. Kolyganova, Vera G. Arzumanian, Kerim Mutig, Mikhail Yu. Samsonov, Svetlana A. Lebedeva

**Affiliations:** 1 Frumkin Institute of Physical Chemistry and Electrochemistry, Russian Academy of Sciences, 119071 Moscow, Russia; 2 The Institute of Pharmacy, Sechenov First Moscow State Medical University (Sechenov University), 119048 Moscow, Russia; victoriababasieva@mail.ru (V.S.B.);; 3 A. E. Favorsky Irkutsk Institute of Chemistry, Siberian Branch of the Russian Academy of Sciences, 664033 Irkutsk, Russia; 4 F. Erismann Institute of Public Health, Department of Microbiology, Virology and Immunology, Sechenov First Moscow State Medical University (Sechenov University), 119435 Moscow, Russia; 5 Mechnikov Research Institute for Vaccines and Sera, 105064 Moscow, Russia; 6 Scientific Center of Genetics and Life Sciences, Sirius University of Science and Technology, 354340 Sirius, Russia; 7 Medical Department, R-Pharm JSC, 123154 Moscow, Russia

**Keywords:** imidazole, *N*-alkenylimidazole, zinc chloride, complexes, antitumor activity, antifungal activity, antimicrobial activity

## Abstract

**Background/Objectives**: Transition metal complexes of imidazoles exhibit a variety of biological activities. This makes them promising metal-based drugs for use in medicine. The aim of this research is to investigate the complexes of zinc(II) with *N*-vinyl, *N*-allyl, and *N*-propargylimidazoles, represented by the formula [ZnL_2_Cl_2_], as potential drug candidates. **Methods**: Structural studies of the obtained complexes were performed using single-crystal X-ray diffraction analysis, IR and NMR spectroscopy. DFT calculations were used to determine structural, electronic and thermochemical parameters of the complexes. QSAR analysis was performed using PASS. The wound-healing and antihypoxic activities were studied *in vivo* using models of wounds and acute hypoxia of various origins. The antimicrobial activity of the complexes was evaluated against *Staphylococcus aureus* Wood 46, *Escherichia coli* M-17, and the yeast fungus *Candida albicans* 927. The cytotoxic activity was tested using several cell lines, including monkey kidney (Vero) cells, human cervical cancer cells (Hep2C and HeLa), human lung carcinoma (A549), and human embryonal rhabdomyosarcoma (RD). **Results**: New complexes of *N*-allylimidazole and *N*-allyl-2-methylimidazole with ZnCl_2_ were synthesized and characterized. All the studied complexes possess diverse biological activities. While the antimicrobial activity was modest, a distinct antifungal activity was observed. The cytotoxicity of the complexes was found to be mainly in relation to Hep2c and RD cell lines. **Conclusions**: Based on the results of QSAR analysis and experimental findings, the diverse biological activities of the compounds indicate that they are promising lead structures for further optimization in drug development.

## 1. Introduction

The development and introduction of promising drugs based on various essential trace elements in the form of their coordination compounds into medicine is one of the most important and challenging issues in modern pharmacology. These complexes possess great potential due to the synergistic effect of their components: essential metals and pharmacologically active small molecules as ligands. As a result, metal complexes often exhibit higher biological activity than the corresponding free ligands [1,2,3,4]. The prospects for using these drugs are quite promising due to their diverse range of targeted actions, low toxicity, ease of dosing, and high efficacy [5,6]. Among essential trace elements, two elements, zinc and copper, are the priority metals for drug development [7]. Zinc is the second most abundant trace element after iron, and it plays a significant role in a wide range of biological processes [8,9]. Zinc’s redox inertness and lack of ligand field stabilization energy provide important advantages for coordination, allowing it to maintain diverse coordination geometries [10]. Additionally, zinc(II) complexes undergo rapid ligand exchange reactions, facilitating Lewis acid activation, enabling them to play a crucial role in DNA cleavage processes [11,12]. Zinc(II) complexes have fewer side effects, even at high concentrations, compared to other metal complexes [13]. This, along with other therapeutic benefits of zinc-based compounds, makes them a more promising alternative to platinum complexes for antitumor therapy [14]. Most of the zinc metal complexes currently under development exhibit more potent antitumor activity than cisplatin, including against cisplatin-resistant cell lines [14,15,16,17].

Metal complexes based on nitrogen-containing heterocyclic compounds, found in both naturally occurring and synthetic physiologically active substances, actively participate in enzymatic processes and have immense biological potential for medical applications. Imidazole derivatives, among nitrogen heterocyclic ligands, are a promising area of research in this field. These derivatives are structural components of many naturally occurring and synthetic compounds that play an important role in enzyme activity and the binding of microelements within living organisms [18]. Imidazole derivatives play a fundamental role in medicinal chemistry [19]. Their unique amphoteric structure and ability to interact with a variety of receptors make them highly effective as broad-spectrum antimicrobial, antifungal, antiviral, and antitumor therapies and treatments for cardiovascular and gastrointestinal disorders [20,21]. Imidazole easily binds with transition metal cations to form various complexes with broad medicinal potential [22]. It has been shown that the biological activity of imidazole derivatives is often enhanced by complexation with zinc(II), in particular ZnCl_2_ [23]. The zinc(II) complexes with imidazoles showed antimicrobial activity against various bacterial and fungal strains. This was attributed to both the imidazole ring and the zinc(II) cation [24,25,26]. Among imidazole derivatives, *N*-alkenylimidazoles have recently gained attention, and a related drug, Acyzol, has been developed. It is based on diacetatobis(*N*-vinylimidazole)zinc(II), which is highly effective in preventing and treating carbon monoxide poisoning, hypoxia, and psoriasis [27]. The successful development of Acyzol has led to active investigations into improved analogs. As a result, a number of *N*-alkenylimidazole metal complexes have been identified, including complexes of *N*-vinyl-, *N*-isopropenyl- and *N*-allenylimidazoles with various zinc salts [28,29,30,31,32,33]. These compounds have demonstrated promising activity against various types of hypoxia. The cobalt chloride complex of *N*-vinylimidazole (Cobazol) has been described as an erythropoiesis-stimulating agent for the treatment of iron deficiency anemia and chemotherapy- and radiation therapy-induced anemia [27]. The reported data clearly show that transition metal complexes of imidazoles with unsaturated substituents have not only pronounced antihypoxic activity but also other beneficial properties, making them promising candidates for use as metal-based drugs in medicine. Recent publications have clearly demonstrated the continued relevance of structural, computational, and biological investigations of transition metal complexes of imidazoles [34,35,36,37]. These studies highlight current trends, such as the integration of structural and spectroscopic studies, QSAR, and quantum-chemical computational analysis with biological evaluation, revealing correlations between the structural and biological properties [38,39,40,41]. However, previous studies often lacked information about the thermodynamics of complex formation and dissociation. In this paper, we focus on the thermodynamics, in conjunction with structural, theoretical, and biological research on zinc(II) chloride complexes with imidazole derivatives containing unsaturated substituents, with the goal of assessing their potential as drug candidates.

## 2. Results and Discussion

### 2.1. Synthesis

**Scheme 1:**
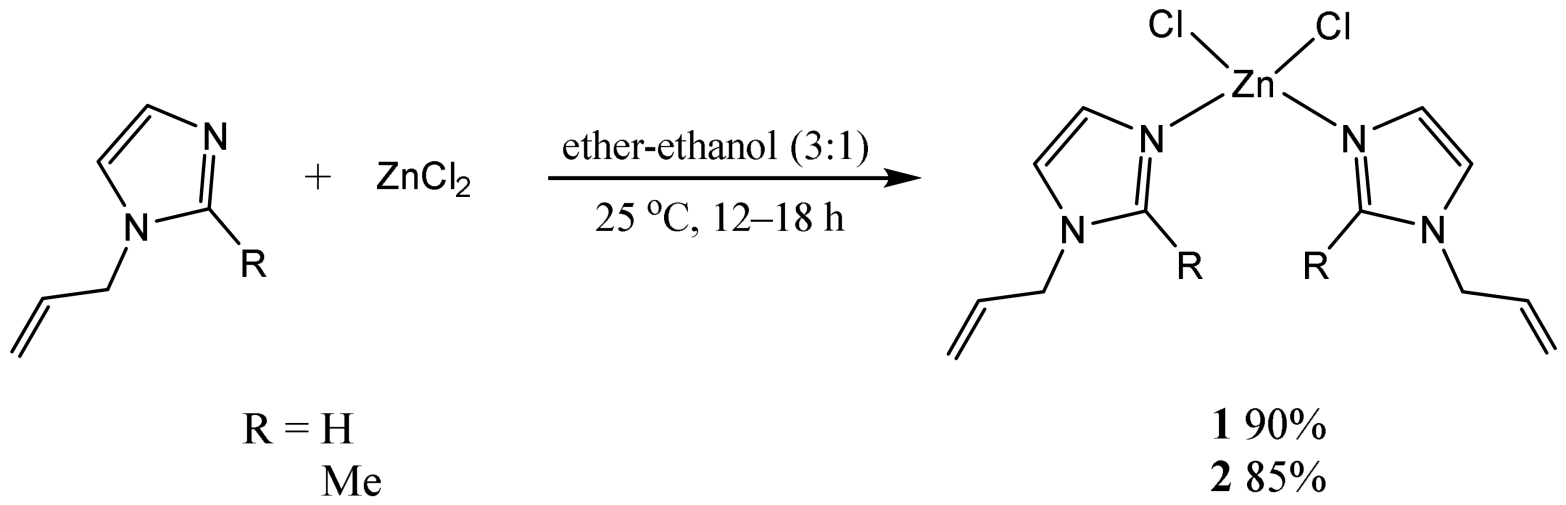
Synthesis of complexes of zinc(II) dichloride with *N*-allyllimidazole (**1**) and *N*-allyl-2-methylimidazole (**2**).

Two new complexes of zinc(II) dichloride with *N*-allyllimidazole and *N*-allyl-2-methylimidazole were synthesized by mixing the ligands and zinc salt in a 3:1 mixture of ether and ethanol at room temperature. Regardless of the ratio of the reagents (L/ZnCl_2_ = (2–4:1), only complexes of the composition [ZnL_2_Cl_2_] were formed (Scheme 1). A slight excess of ligand relative to salt (2.1–2.5:1) ensured the absence of free zinc(II) dichloride in the product, leading to the highest yield (85–90%) of complexes with the best purity. Complexes dichlorobis(*N*-allylimidazole)zinc(II) (**1**) and dichlorobis(*N*-allyl-2-methylimidazole)zinc(II) (**2**) were isolated as air-stable colorless solid products. In addition, two other complexes that were previously reported, dichlorobis(*N*-propargylimidazole)zinc(II) (**3**) [30] and dichlorobis(*N*-vinylimidazole)zinc(II) (**4**) [29], were also synthesized and studied. The substances are soluble in water and organic solvents such as acetone, chloroform, acetonitrile, ethanol and dimethyl sulfoxide and are insoluble in diethyl ether. Complex **2**, which contains an additional methyl group, is less soluble in water than **1**, and it swells in it. All the complexes were completely bench stable in pure form, showing no signs of decomposition.

### 2.2. NMR Spectra

NMR spectra were acquired using CDCl_3_, DMSO-d6, and D_2_O as solvents. The ^1^H NMR spectra of all the samples showed resonances only for protons in the complexes, with no signs of free ligands (Figures S5, S7, S9 and S11). The spectra in D_2_O remained unchanged after a week, confirming the stability of the complexes against hydrolysis to a degree that is consistent with the sensitivity of the NMR method at the relatively high concentration of 10 g/L. Signals of the protons of the imidazole cycle were significantly low-field shifted compared to those of free ligands, due to the electron-accepting effect of the metal cation on the ligand (Table S16). This confirmed the formation of a complex. The resonance of the C^2^H proton in the imidazole ring, attached to the carbon atom between the two nitrogen atoms, was shifted up to 0.7 ppm. The signal of C^2^CH_3_ in **2** was shifted by 0.26 ppm, while other ring protons were shifted by between 0.1 and 0.4 ppm. The protons of the allyl and propargyl groups were only slightly affected due to their lack of conjugation, but the signals of the vinyl group conjugated with the ring were shifted up to 0.5 ppm. The signals of carbon atoms were not significantly shifted from those of the free ligands.

### 2.3. IR Spectra

In the IR spectra of the complexes, the coordination of the nitrogen atom to the metal resulted in a distinct shift in the absorption bands of the imidazole cycle (ν(C=N) conjugated with ν(C=C)) by 10–30 cm^−1^ to the higher frequency, compared to the bands of the free ligand (1506 cm^−1^). As a result, these bands were recorded at 1526 and 1542 cm^−1^ in the IR spectra of complexes **1** and **2**, respectively. In addition, the intensity of the band of valence vibrations of the allyl groups (ν(C=C)) at 1640–1650 cm^−1^ was significantly decreased in the complexes compared to that of free ligands. Medium- and low-intensity absorption bands were also observed in the range of 3142–3114 cm^−1^. These bands are attributed to the ν(=C-H) vibrations of the allyl group and the ν(=C-H) vibration of the heteroaromatic ring.

### 2.4. X-Ray Study

**Figure 1:**
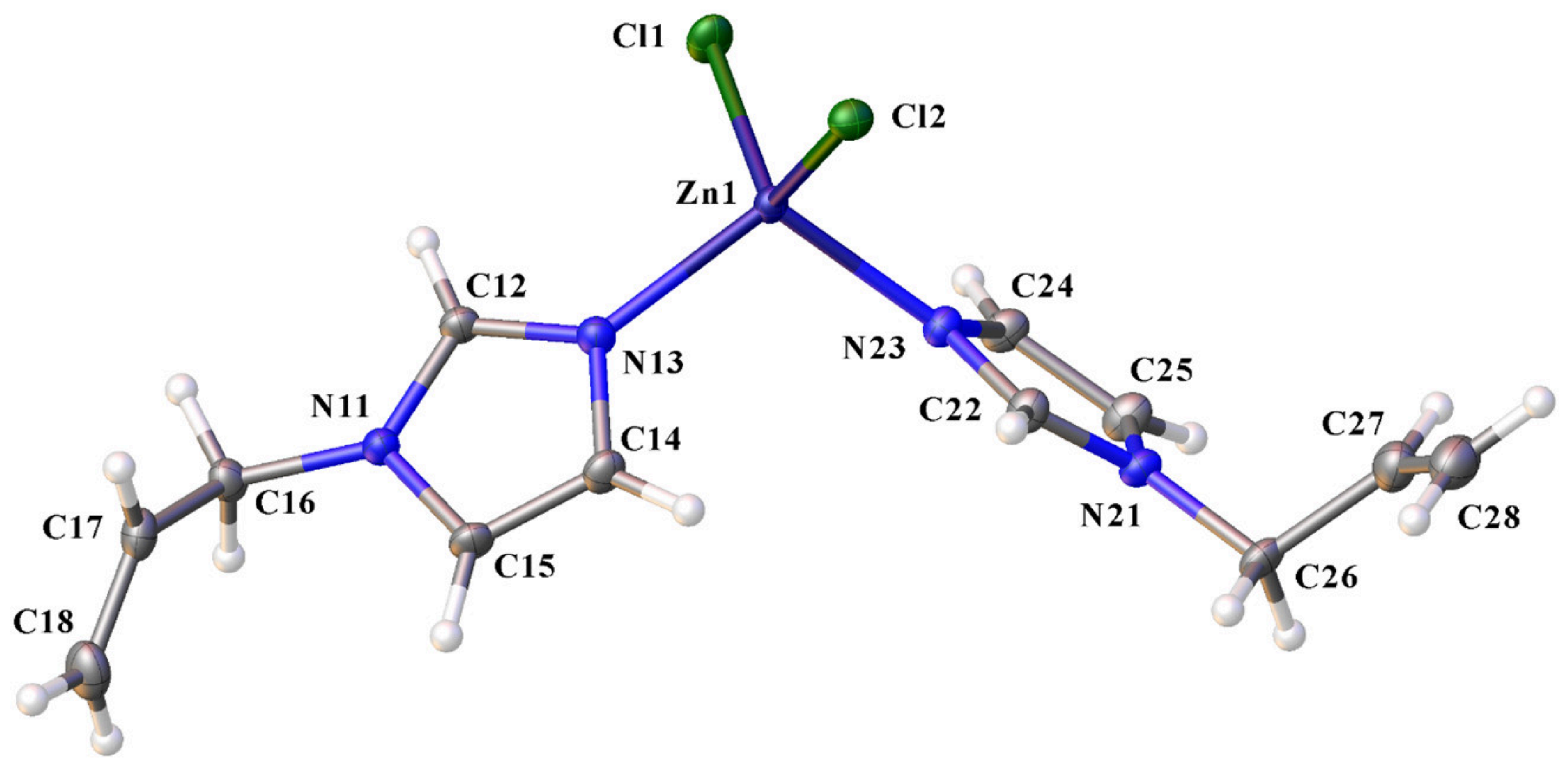
Molecular structure of complex **1**. The ORTEP diagram was generated with displacement ellipsoids at 50% probability. H atoms are shown as spheres of arbitrary radius.

**Figure 2:**
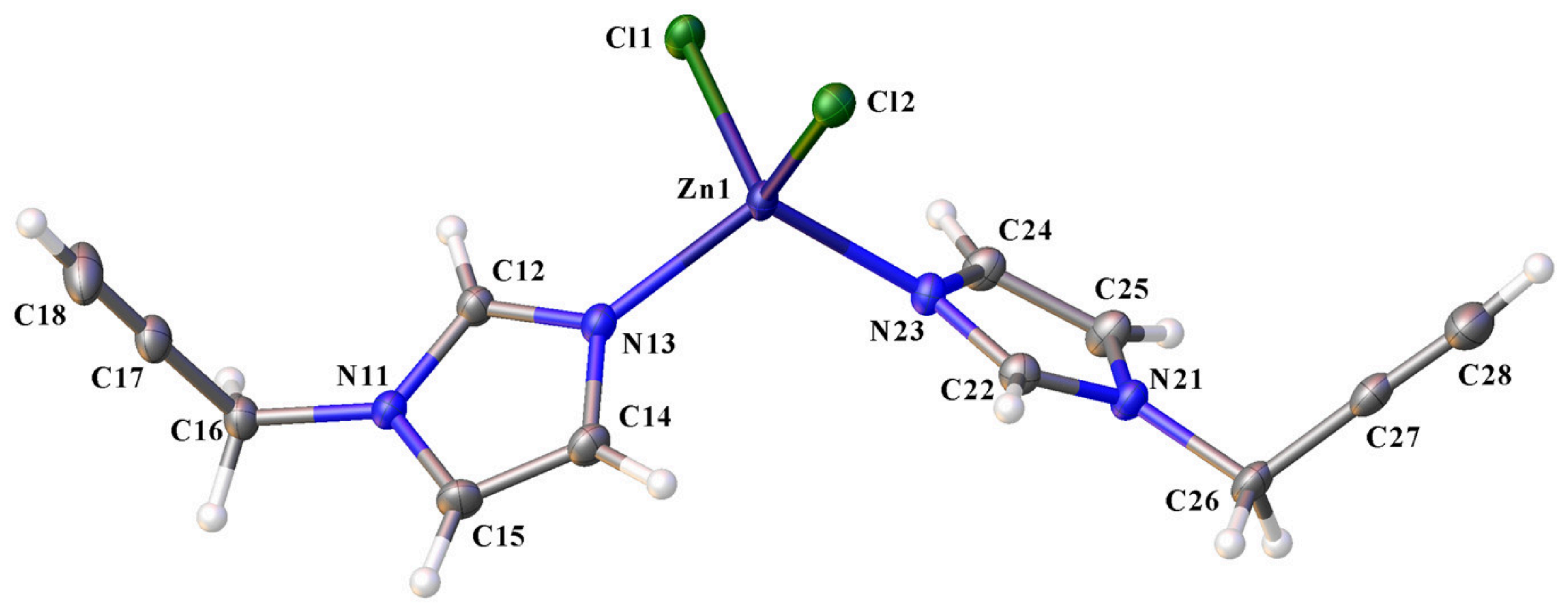
Molecular structure of complex **3**. The ORTEP diagram was generated with displacement ellipsoids at 50% probability. H atoms are shown as spheres of arbitrary radius.

The crystal structures of two complexes, dichlorobis(*N*-allylimidazole)zinc(II) (**1**) and dichlorobis(*N*-propargylimidazole)zinc(II) (**3**), were studied using single-crystal X-ray diffraction analysis. The crystals suitable for diffraction analysis were obtained by slow diffusion of diethyl ether into an acetone solution of the compounds. Both the complexes crystallize in the orthorhombic crystal system with the space group *Pbca* and contain eight molecules per unit cell (Table S1, Figures S1 and S2). Two imidazole ligands and two chloride anions coordinate with the zinc(II) cation, forming a distorted tetrahedral geometry around it. The bond lengths Zn-N, as well as Zn-Cl, are almost equal in pairs, with a difference of 0.01 to 0.02 Å. The Zn-Cl bond lengths (2.25 Å) are significantly shorter than the sum of their ionic radii (2.55 Å), confirming a true coordination bond rather than an electrostatic interaction. The Zn-N bond (2.00 Å) is quite short, corresponding to strong bonding. Two ligands are nonequivalent, differing in conformation: allyl and propargyl substituents are rotated at different angles relative to the imidazole cycle. Allyl substituents are out of the plane of the imidazole rings with torsion angles of the allylic groups C25-N21-C26-C27 and C12-N11-C16-C17 (Figure 1) being 80 and 133°, respectively. Propargyl groups are generally closer to a perpendicular position to the imidazole plane, with torsion angles of −83 and −102° between C12-N11-C16-C17 and C22-N21-C26-C27, respectively (Figure 2). Imidazole rings are also rotated relative to each other, with different torsion angles between the C-N and Zn-Cl bonds. The corresponding torsion angles C14-N13-Zn1-Cl2 and C22-N23-Zn1-Cl2 are 67 and 37°, respectively. The same is true for **3**, with torsion angles of C12-N13-Zn1-Cl1 being 0° and C22-N23-Zn1-Cl2 being 45°. Molecules in the crystal lattice are bonded together through weak hydrogen bonds. These bonds occur between chlorine atoms and all three hydrogen atoms of the imidazole ring (C12H12, C14H14, and C15H15), as well as one hydrogen atom from the methylene group in one of the allyl substituents (C26H26A). The relatively acidic acetylenic hydrogen atom in the propargyl substituents also interacts with Cl through hydrogen bonding. The hydrogen bonds are quite long, with lengths ranging from 2.6 to 2.9 Å (Tables S6 and S11). It should be noted that a different polymorph of **3** has been reported [30]. The two forms differ from each other in terms of molecule shape and crystal packing. The previously reported form has a monoclinic crystal structure with a *P*2_1_/*c* space group.

### 2.5. Quantum-Chemical Calculations of Complexes

The structures and properties of all the complexes in solution were investigated using the DFT method. To make accurate predictions, it is important to select an appropriate functional from the large family of available ones. The Minnesota functionals have been reported to be well-suited for coordination compound calculations [42]. M06L was shown to provide accurate predictions for metal complexes, including zinc complexes [43]. Improved MN15 provides better accuracy for larger systems, including those with noncovalent interactions. It performs exceptionally well for biologically relevant complexes of transition metals, in particular zinc [44]. Therefore, the geometries of the complexes, as well as those of all the precursor molecules, were optimized using the MN15 global-hybrid exchange-correlation functional in conjunction with a split-valence, double-zeta basis set 6-31+G(d,p) with polarization functions on all atoms and diffuse functions on heavy atoms. This combination is well-suited for geometry optimizations, and the calculated parameters correspond well to the experimental data. The structures of the complexes were calculated in aqueous solution using the polarizable continuum model. The calculated bond lengths for the compounds whose crystal structures have been studied are close to those determined by X-ray diffraction (Table S17). All of the calculated structures have a distorted tetrahedral coordination sphere, similar to that seen in crystals (Figures S19–S22) [30].

#### 2.5.1. Thermochemical Parameters of the Complexes

The stability of the complexes was assessed using thermochemical parameters. The energy of the molecules was calculated using a larger high-quality triple-zeta valence basis set 6-311+G(2df,2p) featuring additional polarization functions and diffuse functions for greater precision. The calculated structures and their thermodynamic parameters are presented in Table 1. The enthalpies and Gibbs free energies were calculated for the reaction of complex formation between ZnCl_2_ and the ligands in an aqueous solution according to Equation (1). The zinc salt was considered in the form of an aqua complex ZnCl_2_ × 2H_2_O, as it has been reported that it predominantly exists at concentrations greater than 2 M in this composition [45].

ZnCl_2_ × 2H_2_O + 2L → [ZnL_2_Cl_2_] + 2H_2_O (1)

As can be seen from Table 1, most of the complexes exhibit sufficiently high stabilities in aqueous solution. However, substituents have a distinct influence on the stability of complexes. The lowest stability is inherent in the *N*-vinylimidazole complex, where the carbon–carbon double bond is directly attached to the cycle. Among all the compounds, complex **4** shows the lowest stability, with a formation constant that is three orders of magnitude lower than that of **2**. The reason for this is that the alkenyl substituents on the nitrogen atom act as electron acceptors relative to the imidazole π-electron system, due to the strong -M (negative mesomeric) effect. The electron deficiency of the imidazole ring decreases the electron donor capabilities of the coordinating nitrogen atom, and consequently, the Bronsted and Lewis basicities and ligand strengths of the molecule. Propargyl and allyl derivatives are more stable because the saturated methylene group (CH_2_) between the nitrogen atom and the multiple bond in the allyl and propargyl groups blocks electron conjugation between the imidazole ring and its fragments. Comparing monosubstituted imidazoles with disubstituted ones, it can be seen that the additional 2-methyl group contributes to higher stability. The enthalpy of formation for **2** is about 5 kcal/mol higher than for **1**, due to the +I (positive inductive) effect of the methyl group, which increases the electron density in the heterocycle and increases the ligand’s basicity.

Despite the general thermodynamic stability of the complexes, partial dissociation may occur in aqueous solution. Hydrolytic dissociation constants were calculated based on the thermochemical parameters of partially hydrolyzed complexes (Table 2). These parameters were calculated for the dissociation of one anion from the complex, replacing it with a water molecule according to Equation (2):

[ZnL_2_Cl_2_] + 7H_2_O → [ZnL_2_(H_2_O)Cl]^−^ + [Cl(H_2_O)_6_]^−^ (2)

The hydrolytic dissociation process is an endothermic reaction, and the corresponding constants are low, indicating that the dissociation occurs mostly at low concentrations. The highest dissociation constant, around 0.05 (mol/L)^−1^, is characteristic of **4**, which also has the lowest formation constant. However, there is no general trend in the relationship between formation and hydrolytic chloride anion dissociation constants, as formation constants were calculated for the coordination of ZnCl_2_ with ligands. In the alternative hydrolytic dissociation process, the ligand can dissociate and be replaced by a water molecule in the complex. The thermochemical parameters for the partial dissociation (hydrolysis) of one ligand have been calculated according to Equation (3) (Table 3). The values of enthalpy and free energy of partial hydrolytic dissociation of the ligand are in parallel with the stability of the complexes and the corresponding values of their formation from ZnCl_2_ and free ligands (Table 1). It can be clearly seen that the dissociation of the metal–chloride anion proceeds considerably more easily than metal–ligand. The dissociation of ligands is more endothermic and has lower equilibrium constants than the dissociation of chloride anions. Therefore, it is more likely that anions will dissociate in an aqueous solution, while the ligands will be retained. In confirmation of this, the NMR spectra of the complexes in aqueous solution did not show any signs of ligand dissociation. The signals in the NMR correspond only to the coordinated ligands.

[ZnL_2_Cl_2_] + H_2_O → [ZnL(H_2_O)Cl_2_] + L (3)

However, all the above conclusions were drawn for pure water solutions. Under physiological conditions, the presence of chloride anions suppresses, to some extent, the dissociation of chloride complexes. On the other hand, the presence of competitive ligand binding sites in serum proteins that can bind zinc ions can also lead to the release of free imidazole ligands into solution.

#### 2.5.2. Frontier Molecular Orbitals (FMOs) Parameters

**Figure 3:**
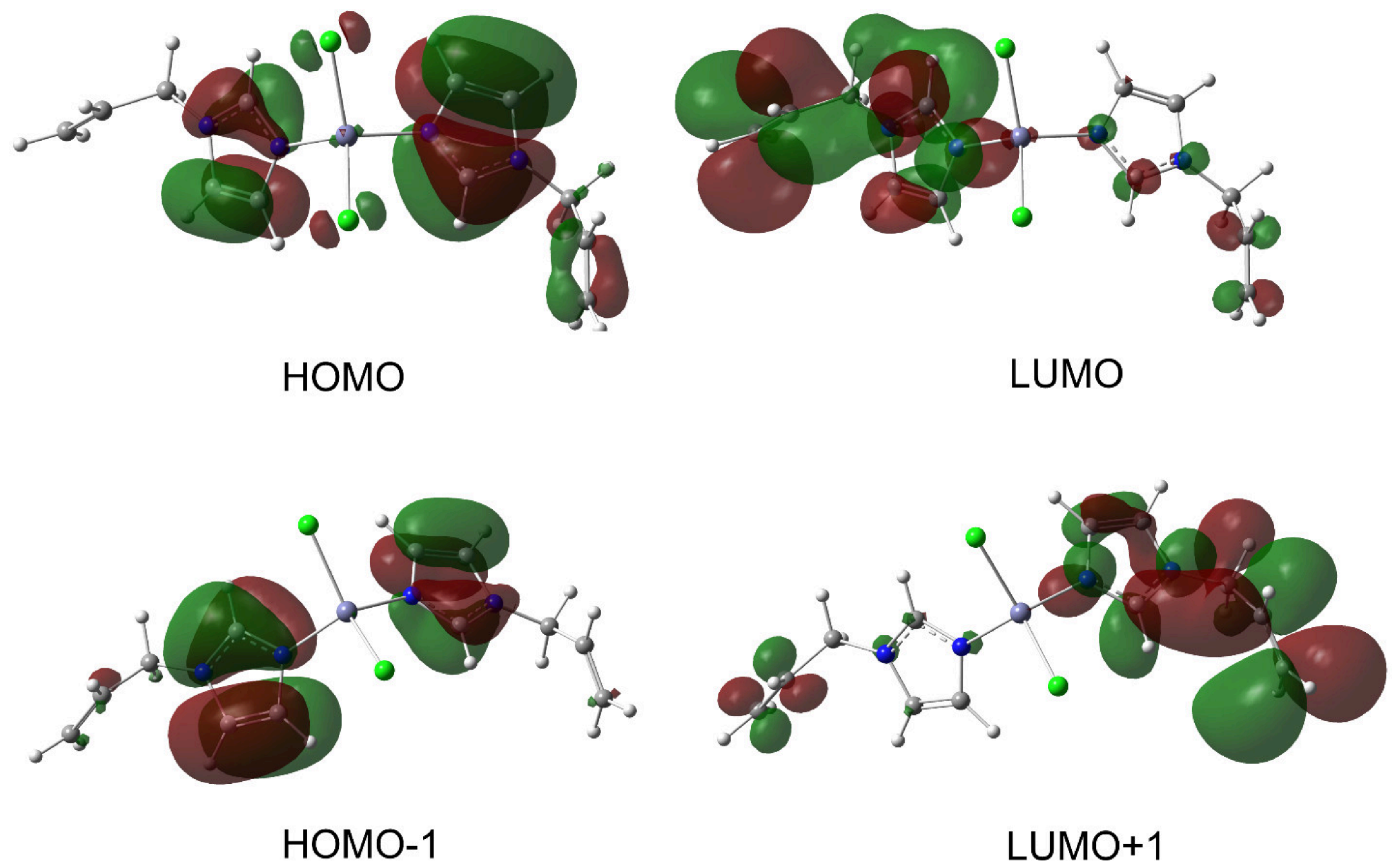
Frontier molecular orbitals plot for complex **1**.

The electronic properties of the complexes can be inferred from their molecular orbital composition and depend largely on the frontier molecular orbitals (FMOs). These orbitals—the highest occupied molecular orbital (HOMO) and the lowest unoccupied molecular orbital (LUMO)—have been calculated, and a representative example of the FMOs for **1** is shown in Figure 3. Other examples can be found in the SI (Figures S23–S25). The frontier molecular orbitals of all the complexes are located mainly at ligand fragments. Due to the symmetry of the two ligands, FMOs consist of two almost degenerate pairs of orbitals: HOMO, HOMO−1 and LUMO, LUMO+1. Orbitals are typically delocalized across both ligands, with one orbital being more centered on one ligand and the other orbital being more centered on the other ligand. However, in some cases, such as in complexes of *N*-allylimidazole, vacant orbitals may not be delocalized, with the LUMO being located on one ligand molecule and the LUMO+1 being located on the other molecule.

Another feature is that HOMO is mostly located at the imidazole ring, while LUMO includes an unsaturated substituent. In complex **1**, LUMO consists mainly of the antibonding orbital of the C-C double bond in the allyl group. In the LUMO, there is a direct overlap of the π-orbitals from the conventionally isolated double bond and the imidazole ring. While the unsaturated substituent, isolated by a methylene group, has a weak effect on the ground state of the complex, it has a significant effect on the excited and anionic states.

Based on the energy levels of FMOs, the electronic parameters of the ligands and their Zn complexes were calculated. Particularly, ionization potentials (I) and electron affinities (A), energy gap (ΔE), the global reactivity descriptors, electronegativities (χ), hardness (η), chemical potentials (µ), global softness (S), electrophilicity (ω) and maximum charge transfer (ΔN_max_) were calculated (Table 4). The dipole moment (*p*) was also calculated based on the total density.

The absolute softness of a molecule refers to its ability to interact with other molecules, such as biological compounds. This parameter of the complexes coincides with ligands and does not vary significantly between different compounds. Therefore, all complexes exhibit similar levels of potential biological activity based on this parameter. Both the electronegativity (χ) and electrophilicity (ω) of the complexes are generally higher than those of the ligands. The electronegativity (χ), electrophilicity indicator (ω) and maximum charge transfer (ΔN_max_) are highest for *N*-alkenyl ligands and their complexes, with **4** having the highest values. The dipole moment (*p*) is the most variable parameter during complexation, increasing 3–4 times. The additional methyl group in the ring significantly enhances the electron-donating ability of the ligand, leading to the highest dipole moment for **2**. The statistical analysis of the correlation of the measured biological activities of the complexes with the electronic parameters is given in Table S20.

#### 2.5.3. Molecular Electrostatic Potential Maps (MEPs)

**Figure 4:**
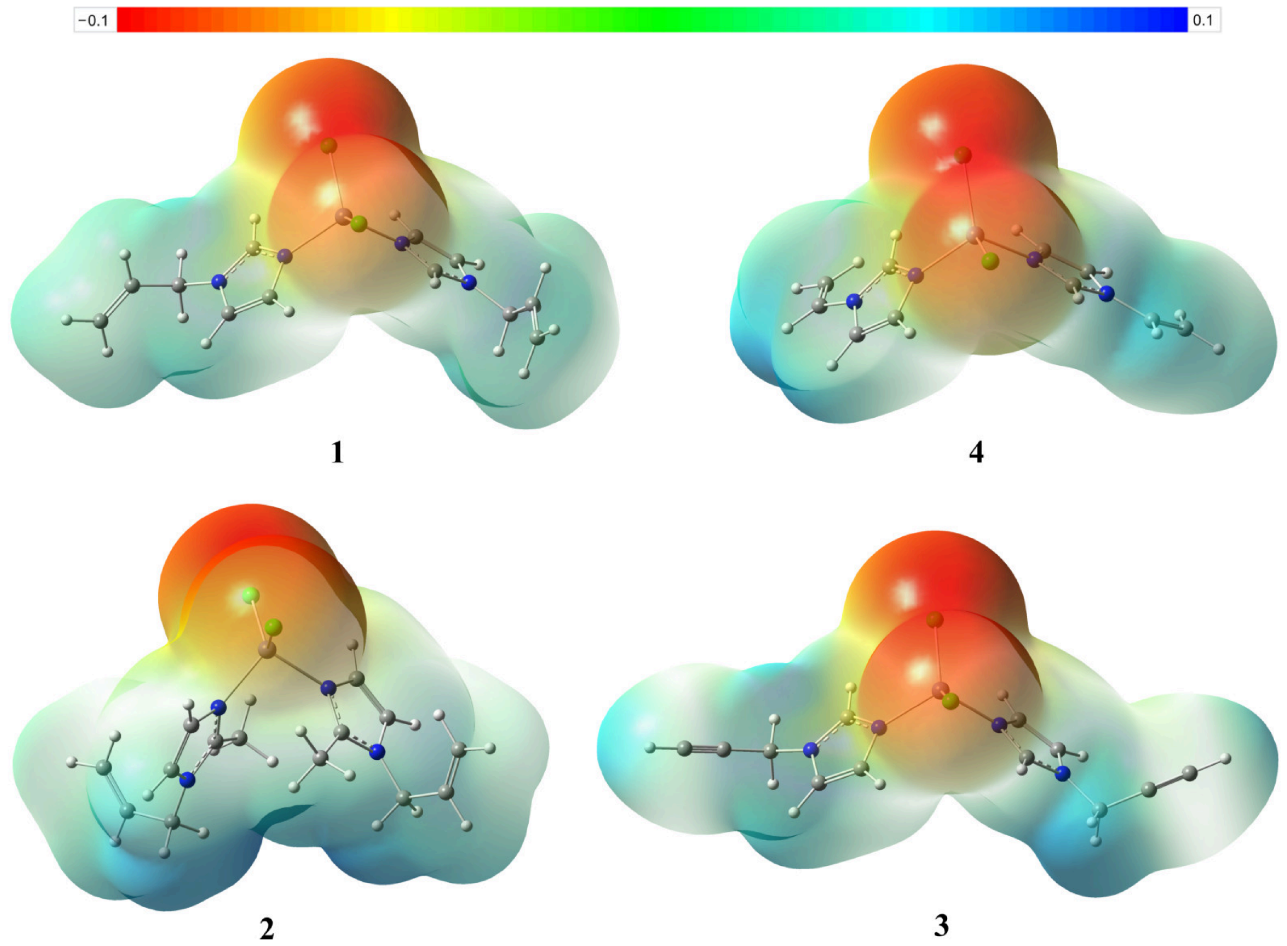
The molecular electrostatic potential surface of the complexes **1**−**4**. The color legend indicates the magnitude of the charge density.

Molecular electrostatic potential (MEP) surfaces were generated from full density of DFT calculations in order to visualize the electronic distribution within molecules. This information can help to identify electrophilic and nucleophilic sites within a molecule. The color of the MEPs corresponds to the electron density, with longer wavelengths corresponding to higher electron densities. MEPs for the complexes (Figure 4) show that electron-rich regions are located around chlorine atoms. The ligand part of the molecules exhibited a moderate amount of electron deficiency. Compounds **1** and **3** resemble each other in their MEPs. Complex **4** has a distinct electron deficiency (represented by a blue-green color) at the *N*-vinyl substituent compared to *N*-allyl in **1**, which also corresponds to the higher electrophilicity of the former (ω = 2.51). A slightly higher electron density (represented by an orange-red color) is observed around the chlorine atoms of both **2** and **4** compared to that of **1**. This higher polarization is also in line with the higher chemical potential of **4**. However, complex **2** shows the highest charge difference, corresponding to its highest dipole moment.

### 2.6. Biological Activity Studies

#### 2.6.1. Antihypoxic Activity

Previously, we demonstrated that diacetatobis(*N*-allylimidazole)zinc(II) has a protective effect against acute hypobaric and acute hypoxic models with hypercapnia, but it is ineffective against hemic and histotoxic hypoxias [28]. Other studies on imidazole complexes of zinc(II) diacetate also showed their antihypoxic effects [46,47]. The observed antihypoxic activity is likely due to improvements in the blood’s oxygen-binding and gas transport functions. Antihypoxic activity of the complexes **1**–**4** was studied in nonlinear male mice weighing 20–22 g using models of acute hypoxia: hemic, histotoxic, and hypoxia with hypercapnia. The antihypoxic effect of the complexes at three different doses (10, 25, 50 mg/kg) was assessed based on the lifespan of mice exposed to acute hypoxia compared to the control group (Table 5). Each group, subjected to one type of hypoxia and a single dose of treatment, consisted of 10 mice. The choice of doses, timing, and routes of administration for the study was based on data on the antihypoxic activity of a previously studied complex of *N*-allylimidazole and zinc(II) diacetate [28]. Complexes **1**, **3**, and **4** did not exhibit antihypoxic properties. However, complex **2** did exhibit some limited antihypoxic activity. The results of this study, along with some previous research, indicate that the antihypoxic efficacy of zinc(II) chloride complexes is lower than that of zinc(II) acetate complexes. This highlights the significant role of the anion in the biological activity of the complexes [30]. The acetate complexes are more lipophilic and are better transported by albumin in blood plasma, and they can more easily penetrate through cell membranes. The cellular uptake of acetate complexes is significantly higher than that of chloride complexes, which provides their higher biological activity. On the other hand, the acetate anion has a much lower ligand strength than the chloride anion. The acetate complexes with zinc(II) are significantly less stable than the corresponding complexes of chlorides, with formation constants several orders of magnitude lower. All these properties suggest that acetate complexes could be considered an efficient zinc delivery vehicle. Upon delivery, their dissociation releases free zinc(II) cations (aqua complexes) and free imidazole ligands as active species. On the other hand, the lower stability and high hydrolytic dissociation constants of acetate complexes, along with the presence of chloride anions in biological media, and the rapid ligand exchange typical of zinc(II) complexes, should lead to eventual substitution of acetate anions by chloride anions. One can expect that this replacement would mitigate the original difference between the chloride and acetate complexes. However, the discrepancy between the expected and observed results suggests that the kinetics of exchange in the biological media is sufficiently low. It is necessary to conduct a special study on the corresponding pharmacokinetics to shed light on this phenomenon. This is an ongoing project, and we will report on it in the future.

#### 2.6.2. Wound-Healing Activity

**Figure 5:**
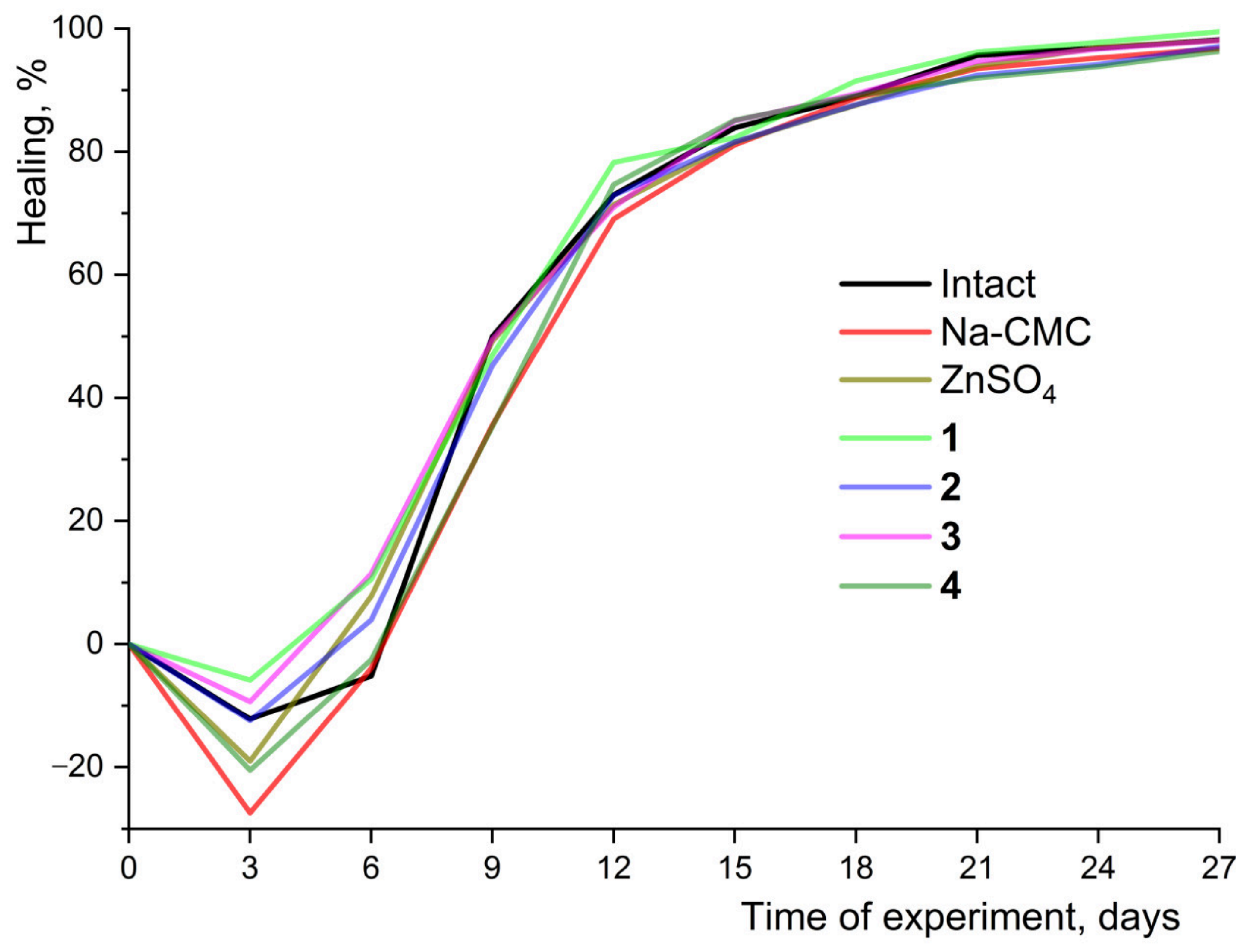
Healing dynamics of a planar wound in rats.

**Figure 6:**
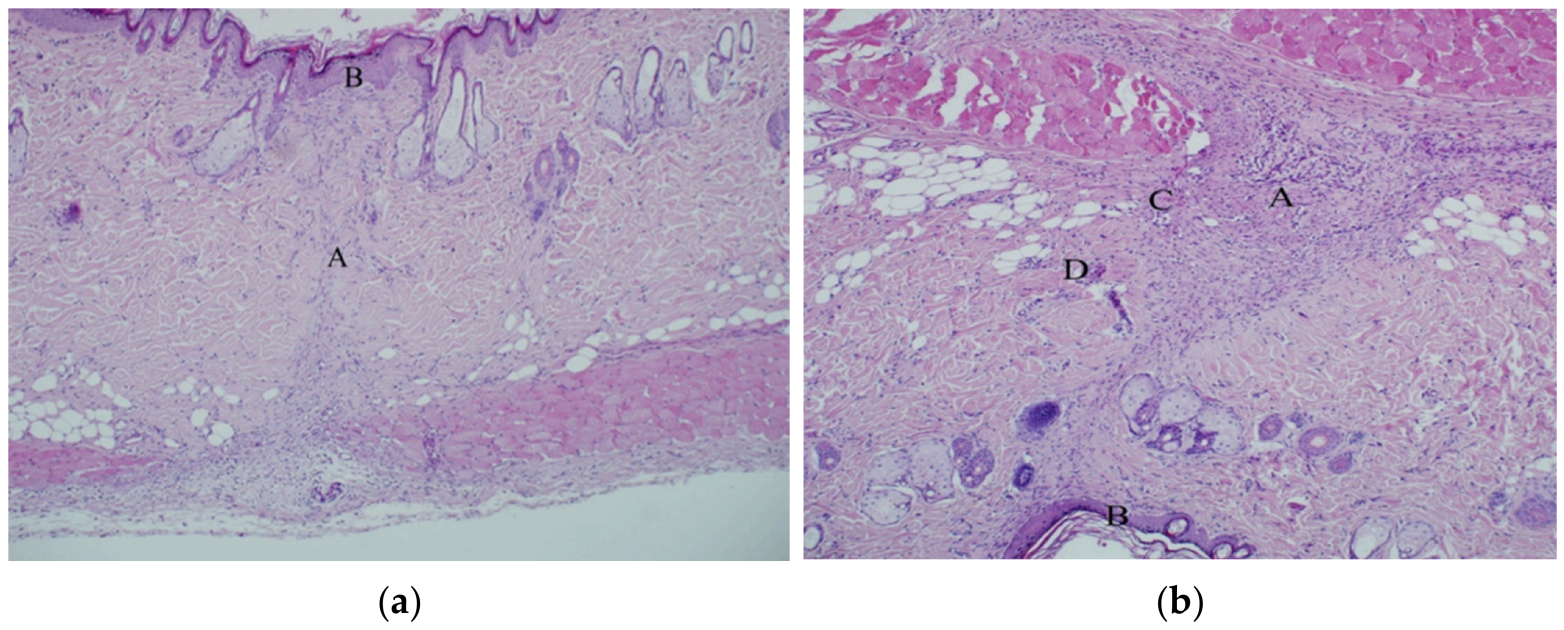
Micropreparations of planar skin wounds in rats. Staining with hematoxylin and eosin. Magnification ×200: (**a**) Control group. Granulation tissue with signs of maturity (A), complete epithelialization (B); (**b**) exposed to **1**. A large amount of granulation tissue (A), complete epithelialization (B), diapedesis, moderate angiogenesis (C), arteriole with thickened wall (D).

Zinc is an essential participant in all stages of wound-healing, as it plays a role in keratinocyte differentiation, proliferation, and has anti-inflammatory and membrane-stabilizing properties [48,49]. Therefore, the study of complexes with *N*-alkenylimidazoles for their wound-healing potential is of great interest. For diacetatobis(*N*-isopropenylimidazole)zinc(II), a significant wound-healing effect has been revealed in models of linear and planar wounds in rats. This effect is likely due to the elimination of hypoxia, improvement of microcirculation, and normalization of free radical oxidation processes [50]. The wound-healing activity of the zinc(II) chloride complexes was evaluated by applying gels containing 1% of the complex to a model of a wound on rats. However, the use of these gels did not significantly reduce the time required for the damaged skin of the rats to heal (Figure 5). This was confirmed by histological examination data, which showed complete wound closure at both the epidermal and dermal levels in both the control and experimental groups of animals by day 28 (Figure 6). These results, along with those from the antihypoxic study, further confirm that chloride complexes have significantly lower activity compared to the corresponding acetate complexes.

#### 2.6.3. Antimicrobial Activity

The antimicrobial effect of zinc has been well documented in numerous studies. Antibacterial activity has been shown for complexes of zinc(II) with amino acids [51], iminopyridines [52], 8-aminoquinoline derivatives [53], and a variety of other compounds and nanocomposites. Given the increasing problem of antibiotic resistance, the use of essential elements in antimicrobial formulations is an area that deserves close attention. Furthermore, the presence of antimicrobial activity against both Gram-positive and Gram-negative bacteria as well as pathogenic fungi in drugs with wound-healing properties is an important benefit, since wound damage to the skin can often lead to wound infection. Imidazole derivatives also have wide antimicrobial activity [54,55]. Therefore, the combination of zinc with imidazole represents a highly promising strategy in this area.

Our studies have previously demonstrated the antimicrobial activity of zinc(II) acetate complexes of *N*-allylimidazole and *N*-isopropenylimidazolyl against various types of bacteria, including Gram-positive *Staphylococcus aureus* 209-P (MIC = 125 μg/mL), Gram-negative *Esherichia coli* ATCC 25922 and *Proteus vulgaris* ATCC 6896 (both with MIC = 250 μg/mL), and *Pseudomonas aeruginosa* ATCC 9027 (with MIC = 500 μg/mL) and weak antifungal activity against yeast fungi *Candida albicans* ATCC 10231 and mycelial fungi *Microsporum canis* 352 (both with MIC = 1000 μg/mL) [50]. Additionally, there was a slight antibacterial effect against *Enterococcus durans* B-603, although this activity (MIC = 250 μg/mL) was inferior to that of the reference antibiotic gentamicin [30].

**Figure 7:**
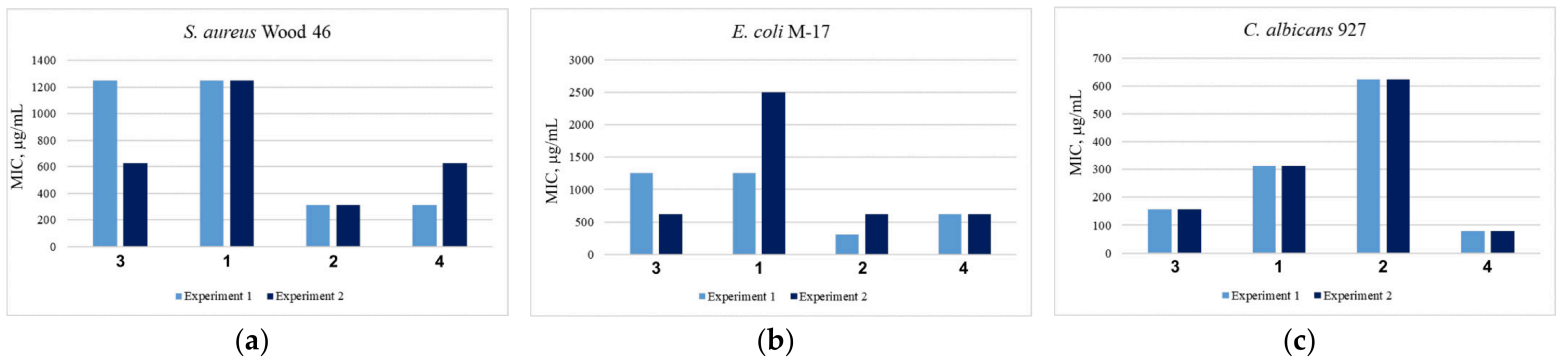
Minimum inhibitory concentrations (MIC) (μg/mL) of the complexes determined by the results of 2 experiments against: (**a**) *Staphylococcus aureus* Wood 46; (**b**) *Escherichia coli* M-17; (**c**) *Candida albicans* 927.

The antimicrobial activity of the complexes was investigated in relation to three strains: the Gram-positive bacterium *Staphylococcus aureus* Wood 46, the Gram-negative bacterium *Escherichia coli* M-17, and the yeast fungus *Candida albicans* 927 (Figure 7). The complexes showed a similar trend, with near-proportional values for both bacterial strains, with the lowest MIC in the range of 300–500 μg/mL for **2** and **4**. The MIC of the studied complexes for *Candida albicans* was significantly lower than for *Staphylococcus aureus* and *Esherichia coli*, reaching 80 μg/mL for **4**. Based on the obtained results, we can conclude that the zinc(II) chloride complexes exhibit a more pronounced antifungal effect than antibacterial activity. The lower antibacterial activity of chloride complexes compared to acetate complexes is in line with other comparative results. However, zinc(II) chloride complexes demonstrated an order-of-magnitude higher antifungal activity. Generally, the antifungal properties of zinc(II) complexes can be explained by the significant role of zinc in maintaining homeostasis and physiological and metabolic processes in fungi. Zinc is essential for enzymes, such as superoxide dismutase and metalloprotease, which are crucial for the virulence and survival of fungal pathogens inside host cells. Additionally, zinc-binding proteins play a role in transcription regulation via different zinc finger transcription factors in *Candida albicans* [56]. The MICs for *Candida albicans* correlate well with the thermodynamic stability of the complexes. Lower stability corresponds to a lower MIC and, consequently, higher antifungal activity. One possible reason for this could be the intervention of dissociated species, such as imidazole ligands and Zn(II) cations, in the zinc(II) coordination equilibrium, thus disrupting zinc regulation in fungi. The electronic properties of the studied complexes and their FMO-based parameters do not correlate well with antimicrobial activity. However, among all the parameters, there is some correlation of MICs for *Candida albicans* with electrophilicity and softness. The most active **4** has the highest electrophilicity and softness parameters. The activity of antifungal drugs has been reported to correlate with FMO parameters [57]. The correlation of activity with both dissociation constants and electronic parameters suggests that the interaction between complexes and fungi may occur through both original and partially dissociated species. The complexes can act by themselves and function as sources of zinc(II) and imidazole ligands, both of which possess antifungal properties. The antifungal properties of nitrogen-containing heterocyclic ligands have been previously demonstrated based on their zinc(II) coordinating ability [58,59].

#### 2.6.4. Antitumor Activity

In addition to antimicrobial properties, zinc has also been shown to have cytotoxic tumor-suppressing effects. This makes it a promising potential therapeutic agent. To date, several zinc(II) complexes have been synthesized and studied, demonstrating antitumor activity. These include pyridine and thiazole derivatives [60], isoquinoline [61,62], oxoaporphine [63], *N*-heteroaromatic selenosemicarbazone metal complexes [64] and others. Imidazole derivatives were widely recognized in medicinal chemistry for their broad spectrum of antitumor properties [65]. They are the core structural component in several established clinical therapies and emerging experimental cancer drugs [66].

The antitumor activity of zinc(II) chloride complexes was assessed using cell lines from the African green monkey kidney (Vero), human cervical cancer cells Hep2C and HeLa, human lung carcinoma A549 cells, human embryonal rhabdomyosarcoma (RD cells), human T-lymphocytic leukemia cell line (MT-4), and acute T-cell leukemia (Jurkat). The antitumor activity of the complexes is expressed in the form of a heat map (Table 6).

None of the studied compounds exhibited a toxic effect on the Vero cell line, which was used as a non-tumor reference (the CC50 value was greater than the selected concentration range). The Hep2c and RD cell lines were found to be the most sensitive to the compounds’ action. At the same time, complex **1** was found to be the most toxic compound for both cell cultures, with CC50 values of 131 ± 33 μM and 69 ± 8 μM for Hep2c and RD, respectively.

The antitumor activity of the complexes is not well correlated with FMO parameters, with the exception of a weak, proportional correlation of CC50 values with electronegativity and hardness. There is no clear correlation with formation constants, but there is a relationship with the hydrolytic dissociation constant of one ligand molecule. The complexes with the highest dissociation constants, both **1** and **2**, correspond to the highest antitumor activities. These results suggest that the complexes may act as sources of other active species, such as imidazole ligands and zinc(II) monoaqua complexes, which can more easily coordinate with intracellular ligands like enzymes and DNA, disrupting their functions. The studies of antitumor activity of imidazolyl-zinc(II) complexes reported that they can act as multifunctional anticancer agents that can induce apoptosis, disrupt metabolism, cause oxidative stress, and inhibit cancer-promoting enzymes [67]. For instance, liposomal zinc complexes cause DNA fragmentation and activate the DNA damage response, both precursors to apoptosis [68]. However, the detailed mechanism of action, including the identification of active species, needs to be studied.

### 2.7. QSAR Study

#### 2.7.1. PASS Analysis

The PASS (Prediction of Activity Spectra for Substances) analysis was used to predict the biological activities of the complexes. Although the model was predominantly trained on organic compounds and its application to metal-containing compounds is uncertain, we can evaluate the general prediction applicability of the model beyond the training set in this case. Due to the lability of zinc(II) complexes and the propensity for dissociation and exchange of anions, the key persistent components of the complexes are the ligand and the zinc(II) cation. In addition, the revealed correlations between dissociation constants and biological activities suggest that the active form may be an aqua complex, rather than a chloride complex. Therefore, more general anion-independent structures were used in the analysis. The results of the PASS analysis list the predicted activity spectra (Pa) for the most probable biological activities. According to the PASS data (Table 7), all the complexes exhibited relatively high probabilities for several common biological activities, including anti-atherosclerosis, antieczematic, chloride peroxidase inhibition, antineoplastic, Cl-transporting ATPase inhibition, fatty-acyl-CoA synthase inhibition, 27-hydroxycholesterol-7-alpha-monooxygenase inhibition, phthalate 4,5-dioxygenase inhibition, and glucan endo-1,6-beta-glucosidase inhibition. Among the compounds, anti-atherosclerosis, antieczematic and chloride peroxidase inhibition properties showed the highest probabilities (Pa), indicating that these compounds have the structural characteristics necessary for these activities. The probabilities of inactivity (Pi) for the corresponding properties were very low, mostly less than 1%. As shown in Table 7, complex **1** had the highest predicted probabilities of being antiatherosclerotic (Pa = 0.88) and antieczematic agents (Pa = 0.84). They also had good potential as chloride peroxidase inhibitors and antineoplastic agents. *N*-vinylimidazole complexes had a high propensity to act as a chloride peroxidase inhibitor (Pa = 0.86), while *N*-propargylimidazole complexes were potentially better antineoplastics. Among all compounds, *N*-allylimidazole had the highest probability values for most potential biological activities.

Importantly, the experimental observation of antitumor activity in the complexes aligns with their predicted antineoplastic potential, validating the PASS output. Additionally, the lack of antioxidant effects is consistent with the low Pa values in the corresponding prediction data. The lower Pa value for antimicrobial compared to antifungal activity also aligns with what was observed in experiments. Therefore, the PASS analysis accurately enough anticipated the major pharmacological trends of the complexes, supporting its use as a screening tool for identifying promising compounds prior to more extensive biological testing.

#### 2.7.2. Assessment of Absorption, Distribution, Metabolism and Excretion (ADME)

The most important physicochemical properties, pharmacokinetics, drug-likeness and medicinal chemistry friendliness, such as absorption, distribution, metabolism and excretion (ADME), were assessed with the SwissADME web-based software. All the compounds are characterized by high gastrointestinal absorption (according to the white of the BOILED-Egg) and bioavailability score of 0.55 (Abbott Bioavailability Score: Probability of F > 10% in rats [69]). Basic physicochemical properties and pharmacokinetic parameters are shown in Table 8.

## 3. Materials and Methods

### 3.1. General

NMR spectra were recorded using a Bruker Avance III 600 spectrometer (Bruker BioSpin, Billerica, MA, USA) (600.13 or 150.90 MHz for 1H and 13C, respectively, in CDCl_3_, DMSO-d6 and D_2_O (99%). All measurements were carried out at 303 K. FTIR spectra for complexes were run on a Bruker Vertex 70 instrument (Bruker Optics, Billerica, MA, USA). Melting points (uncorrected) were measured on a Kofler micro hot-stage apparatus. Elemental analyses for C, H, and N were performed on a Flash EA 1112 CHNS-O/MAS analyzer (Thermo Scientific, Milan, Italy). The Cl content was determined by a method involving the mineralization of samples according to Sheniger, with subsequent titration of chlorides by Hg(II). *N*-allylimidazole and allyl bromide were purified by vacuum distillation. All the other chemicals were purchased from commercial suppliers and used without further purification. Solvents were purified according to a standard protocol.

### 3.2. Syntheses

*N*-Allyl-2-methylimidazole was prepared from 2-methylimidazole and allyl bromide according to the described procedure [72] with minor modifications. Solvents were dried and distilled according to the standard methods. Complexes **3** [29] and **4** [30] were prepared according to the reported procedures.

Dichlorobis(*N*-allylimidazole)zinc(II) (**1**)

ZnCl_2_ (0.63 g, 4.6 mmol) was added portion-wise to a stirred solution of *N*-allylimidazole (1.20 g, 11.1 mmol) in an ether–ethanol (3:1) mixture (40 mL). The suspension was stirred at ambient temperature for 18 h. The white precipitate formed was filtered, washed with ether and dried under vacuum. Yield: 1.46 g (90%), m.p. 130–131 °C. Anal. Calc. for C_12_H_16_Cl_2_N_4_Zn: C, 40.88; H, 4.57; Cl, 20.11; N, 15.89. Found: C, 40.65; H, 4.46; Cl, 20.14; N, 15.45%. IR (selected bands, KBr, cm^−1^): 1647 (C=C_allyl_), 1526 (C=C_imidazole_, C=N). ^1^H NMR, CDCl_3_, δ (ppm): 8.02 (br. s, 1H, C^2^H), 7.21 (br. s, 1H, C^5^H), 7.01 (t, 1H, J = 1.5 Hz, C^4^H), 5.97 (m, 1H, CH_2_CH=C), 5.40 (d, 1H, J = 10.2 Hz, CH=CH_cis_), 5.32 (d, 1H, J = 17.0 Hz, CH=CH_trans_), 4.62 (d, 2H, J = 5.5 Hz, NCH_2_). ^13^C NMR, CDCl_3_, δ (ppm): 138.0 (C^2^H), 131.1 (CH=CH_2_), 127.6 (C^5^H), 120.6 (C^4^H), 120.0 (CH=CH_2_), 50.5 (NCH_2_).

Dichlorobis(*N*-allyl-2-methylimidazole)zinc(II) (**2**)

Complex **2** was prepared by the same procedure as complex **1** from ZnCl_2_ (0.56 g, 4.1 mmol) and *N*-allyl-2-methylimidazole (1.20 g, 9.8 mmol) in an ether–ethanol (3:1) mixture (25 mL) for 12 h, yielding 1.32 g (85%) of **2** as a colorless powder, m.p. 125–127 °C. Anal. Calcd for C_14_H_20_Cl_2_N_4_Zn: C, 44.18; H, 5.30; Cl, 18.63; N, 14.72. Found: C, 44.32; H, 5.21; Cl, 18.80; N, 14.63. IR (KBr, cm^−1^): 1642 (C=C_allyl_), 1542, (C=C_imidazole_, C=N_imidazole_). ^1^H NMR, CDCl_3_, δ (ppm): 7.13 (d, 1H, J = 1.5 Hz, C^5^H), 6.88 (d, 1H, J = 1.5 Hz, C^4^H), 5.91 (m, 1H, CH_2_CH=C), 5.34 (d, 1H, J = 10.3 Hz, CH=CH_cis_), 5.11 (d, 1H, J = 17.1 Hz, CH=CH_trans_), 4.52 (d, 2H, J = 5.5 Hz, NCH_2_), 2.61 (s, 3H, CH_3_). ^13^C NMR, D_2_O, δ (ppm): 145.64 (C^2^), 131.39 (CH=CH_2_), 121.65 (C^4^), 121.37 (C^5^), 118.26 (CH=CH_2_), 49.03 (*N*-CH_2_), 10.71 (CH_3_).

### 3.3. Crystal Structure

Single crystals of dichlorobis(*N*-allylimidazole)zinc(II) (**1**) and dichlorobis(*N*-allyl-2-methylimidazole)zinc(II) (**3**) were obtained by slow diffusion of diethyl ether into an acetone solution. X-ray single-crystal diffraction analysis was performed with a Bruker KAPPA APEX II area-detector diffractometer (Bruker AXS, Madison, WI, USA). The crystals were kept at 100(2) K during data collection. The experimental intensities were corrected for absorption using the SADABS program [73]. The structures were solved by the intrinsic phasing method in SHELXT [74] and refined by the full-matrix least-squares method SHELXL [75] on *F*^2^ for all data in the anisotropic approximation for all non-H atoms. Hydrogen atoms were placed geometrically and refined using a riding model with *U_iso_* constrained at 1.2–1.5 times *U_eq_* of the carrier C or N atoms, except for the terminal CH groups of the propargyl fragments. These H atoms were located from a difference Fourier map and refined without any limitations.

The crystal data, data collection and refinement parameters are given in Table S1 (ESI). Crystallographic data have been deposited with the Cambridge Crystallographic Data Centre, depositions CCDC 2547382-2547383.

### 3.4. Quantum-Chemical Calculations

The quantum-chemical calculations were performed using the Gaussian 16 software package [76] with density functional theory (DFT). The structures of the complexes as well as all the precursors were optimized using the MN15 global-hybrid exchange-correlation functional [77] in conjunction with a split-valence, double-zeta basis set with diffuse functions on heavy atoms 6-31+G(d,p). The single-point energy calculations were performed using a high-quality triple-zeta valence basis set with diffuse functions on heavy atoms 6-311+G(2df,2p). The solvent effects were accounted for by the polarizable continuum model (PCM).

### 3.5. Assessment of Biological Activity via QSAR

#### 3.5.1. PASS Analysis Method

Prediction of Activity Spectra for Substances (PASS) analysis was performed to estimate the possible biological activities of compounds using the PASS Online web server (way2drug.com/PassOnline) using PASS Online version 2.0 [78]. The software was developed and maintained by the Department for Bioinformatics, Laboratory for Structure-Function Based Drug Design, Institute of Biomedical Chemistry (IBMC). The method is based on the structure–activity relationship (SAR) principle, which assumes that similar chemical structures exhibit similar biological properties [79]. Prediction is based on the analysis of structure–activity relationships for more than 250,000 biologically active substances, including drugs, drug candidates, leads and toxic compounds. The software utilizes Multilevel Neighborhoods of Atoms (MNA) descriptors derived from two-dimensional molecular structures to encode atomic environments and functional connectivity within the molecule. Each predicted activity is characterized by two parameters: Pa (Probability of Activity)—the likelihood that a compound will exhibit a specific biological effect, and Pi (Probability of Inactivity)—the likelihood that the compound will not exhibit this effect. Predictions were automatically generated for over 4000 biological endpoints contained in the PASS database. Only activities with Pa significantly exceeding Pi were selected for further consideration, as they correspond to the most probable biological functions for the given structure.

#### 3.5.2. Absorption, Distribution, Metabolism and Excretion (ADME)

The most important physicochemical properties, pharmacokinetics, drug-likeness and medicinal chemistry friendliness such as absorption, distribution, metabolism and excretion (ADME) were assessed with the SwissADME web-based software (https://www.swissadme.ch/) [80]. The method computes physicochemical descriptors and predicts the ADME parameters, pharmacokinetic properties, drug-like nature and medicinal chemistry friendliness of small molecules. The software was developed and maintained by the Molecular Modeling Group of the Swiss Institute of Bioinformatics (SIB).

### 3.6. Biological Studies

All animal experiments were approved by the Local Bioethics Committee of Sechenov First Moscow State Medical University (Sechenov University, Moscow, Russia), protocol no. 03-26 of 6 February 2026. The conditions for animal housing and handling complied with the principles of the Declaration of Helsinki on the humane handling of animals. The study protocol was designed to minimize animal suffering and decrease the number of experimental animals according to the 3R (Reduction, Replacement, Refinement) principle, described in Good Laboratory Practice (GLP) guidelines. Animals were housed in individual cages under stable environmental conditions, humidity (50–70%), and temperature (20 ± 2 °C) in a room with a 12/12 h reversed light/dark cycle. Rats received food and drinking water ad libitum. Surgical procedures were performed under general anesthesia using intramuscular injection of Zoletil^®^ (Virbac, Carros, France) at a dose of 5 mg/100 g of body weight. Animals were humanely euthanized by low flow rates of 100% carbon dioxide in a CO_2_-box.

#### 3.6.1. Antihypoxic Activity Assessment

Antihypoxic activity was studied in nonlinear male mice weighing 20–22 g using models of acute hypoxia: hemic, histotoxic, and hypoxia with hypercapnia. Acute hemic hypoxia was modeled by injection of sodium nitrite at a dose of 300 mg/kg intraperitoneally (i.p.). Acute histotoxic hypoxia was induced by injection of 0.2% sodium nitroprusside solution at a dose of 20 mg/kg, i.p. Acute hypoxia with hypercapnia was induced by placing each animal in a 250 mL glass vial with an air-tight lid. Zinc(II) complexes were dissolved in water for injection, and experimental mice were injected at doses of 10, 25, and 50 mg/kg i.p. 1 h prior to the onset of acute hypoxia. Control animals were simultaneously injected with an equal volume of solvent (water for injection) in the same way. The antihypoxic effect of the substances was assessed by the lifespan of mice in minutes. The experiments used 10 biological replicates.

#### 3.6.2. Wound-Healing Activity Assessment

To replicate the model of a planar skin wound in 42 rats, both male and female, weighing 250–320 g under anesthesia, hair and undercoats were trimmed in an area on the back that was inaccessible to licking. After this, a skin flap with an area of 707 mm^2^ was cut out, including subcutaneous fat. The skin defects were left exposed throughout the observation period. The healing of wound injuries in rats was evaluated by periodically taking wound patterns on tracing paper and weighing them on analytical scales. Local conservative wound treatment was performed in an open manner (without dressing) by evenly applying test substances to the wound surface once a day for 28 days. The wound area was measured twice a week. The average mass of the “wound area” was calculated by weighing a thick paper on an analytical scale, the dimensions of which corresponded in size to the wound.

Based on the measurements taken, the percentage of wound area reduction was calculated using the following formula:

(S − Sn)/S × 100%,

where S is the initial wound area; Sn is the wound area on the measurement day [50,81].

Visually, wound healing was assessed by changes in wound shape, marginal epithelialization, the appearance of granulation tissue, reduced exudate, and formation of a dry scab. Measurements were performed in a blinded manner, and inter-observer variability was assessed.

The experimental animals’ wounds were treated with 200 mg of sodium carboxymethyl cellulose (Na-CMC), which is a hydrophilic base used to create the gel, and 1% gel of zinc(II) complexes based on Na-CMC. 1% zinc(II) sulfate gel based on Na-CMC served as a reference for comparison. In intact animals, wounds healed “naturally” without intervention. After 28 days, the animals were euthanized using a CO_2_ chamber. Material for histological analysis was collected by excising soft tissues and the adjacent edges of the wounds to the fascia. The samples were then stained with hematoxylin and eosin and mounted on cover glass. Visual inspection was performed and photographs of tissues were taken using a Leica DM1000 microscope (Leica Microsystems, Wetzlar, Germany) equipped with a digital camera. Complete epithelialization was assessed by the following criteria: absence of epidermal defects, restoration of epidermal stratification and epidermal barrier continuity.

#### 3.6.3. Antimicrobial Activity Assessment

Antimicrobial activity was determined using the broth microdilution method in round-bottomed 96-well plastic plates (Lenpolymer, Saint-Petersburg, Russia). As test microorganisms, we used the Gram-positive bacterium *Staphylococcus aureus* Wood 46, the Gram-negative bacterium *Escherichia coli* M-17, and the yeast fungus *Candida albicans* 927 (from the collection of the Mechnikov Research Institute of Vaccines and Sera, Moscow, Russia). Microbes were grown until the end of the exponential growth phase on solid nutrient media: glucose-peptone-yeast (YPD) for yeasts and Nutrient Agar for bacteria. Bacteriostatic and fungistatic effects were determined as the minimum inhibitory concentration (MIC) that suppressed microbial growth.

Zinc(II) complexes were dissolved in sterile saline solution (5 mg/mL), and 100 μL of this solution was added to the first cells of the plate. An additional 100 μL of saline solution was added to the remaining cells. After that, 100 μL of zinc(II) complex solution was added to the second cell, and the mixture was mixed. Then, 100 μL was transferred to the next cell, and this process was repeated until all cells had been treated. At the end, 100 μL liquid nutrient media was added to each cell, and the plates were incubated at 32 °C for yeast and 37 °C for bacteria for one day. Each compound was tested in two replicates.

#### 3.6.4. Antitumor Activity Assessment

The adherent cell cultures used were established cell lines of African green monkey kidney (Vero), human cervical cancer cell lines Hep2C and HeLa, human lung carcinoma A549 cells, and human embryonal rhabdomyosarcoma (RD) cells. All the cell lines were obtained from the collection of the Chumakov Federal Scientific Center for Research and Development of Immune-and-Biological Products (Chumakov FSC R&D IBP RAS (Institute of Poliomyelitis)). Cells were cultured in DMEM supplemented with 5% fetal bovine serum (FBS) (Invitrogen, Logan, UT, USA) and a mixture of penicillin 55 U/mL and streptomycin 55 μg/mL (LLC R&P “PanEco”, Moscow, Russia).

The suspension cell cultures used were the human T-lymphocytic leukemia cell line (MT-4) and acute T-cell leukemia (Jurkat). RPMI-1640 medium supplemented with L-glutamine, 5% FBS, and gentamicin (10 μg/mL) was used as the nutrient medium. Cell cultures were maintained at 36.5 °C in an atmosphere containing 5.0 ± 0.5% CO_2_.

Zinc(II) complexes were dissolved in DMSO and then added to the cells. Cytotoxic activity of the complexes was assessed using a fluorimetric resazurin assay in 96-well plates. Fluorescence readings were detected at Ex 560 nm/Em 590 nm using a Fluoroskan instrument (Thermo Fisher Scientific, Vantaa, Finland).

#### 3.6.5. Statistical Data Processing

Statistical data processing was performed using Student’s *t*-test using the IBM SPSS Statistics 23 statistical software package. The sample size was chosen arbitrarily, and after measuring, a power analysis was performed to verify and justify the selected size. For the variation series of the sample, the arithmetic mean (M) and its standard error (m) were calculated. To check the normality of the data, the Shapiro–Wilk test was used. As the data had a near-normal distribution, the significance of differences between experimental groups was determined using one-way analysis of variance (one-way ANOVA), followed by multiple comparisons with the Student’s *t*-test with the Bonferroni correction. For the standard *t*-test, the differences were considered statistically significant at *p* < 0.05. Statistical calculations of the data obtained as a result of fluorimetry were performed using OriginPro 8.1 software (Originlab Corporation, Northampton, MA, USA). The calculation curves for the semitoxic concentration of CC50 were constructed using the nonlinear curve fit function based on the data obtained for each experiment. The obtained CC50 values were averaged between experiments with the calculation of the mean and standard deviation (mean ± SD).

## 4. Conclusions

This investigation, combining computational and experimental approaches, has firmly established the therapeutic potential of the complexes of *N*-alkenylimidazoles with ZnCl_2_. All the compounds were characterized by computationally predicted high gastrointestinal absorption and bioavailability. Among the bioactivities tested, the toxic effect against *Candida albicans* fungi and Hep2C and RD cell lines allowed us to consider these complexes as possible candidates for the development of potential antifungal and antitumor drugs. The complexes of *N*-allylimidazole and *N*-allyl-2-methylimidazole showed higher toxicity compared to the *N*-vinyl- and *N*-propargylimidazole complexes, which is in inverse proportion to their electronegativity and hardness. While the antimicrobial activity of all the complexes was moderate, the complexes of *N*-vinylimidazole and *N*-propargylimidazole exhibited sufficient antifungal activity. The trend in antifungal activity is directly proportional to the electrophilicity, softness and chemical potential of the molecules. The QSAR study of the complexes, in particular, PASS analysis, anticipated the general biological trends of these compounds, which were consistent with experimental results. The concordance between the experimental findings and computational predictions, especially in terms of the observed antitumor activity of the compounds, confirms the predictive power of PASS analysis for early screening of potential compounds. PASS analysis also identifies the complexes as possible agents for anti-atherosclerosis, anti-eczema, and chloride peroxidase inhibition, which need to be tested experimentally. The observed spectrum of biological activity of zinc(II) chloride complexes has been found to be significantly different from that of corresponding zinc(II) acetate complexes. This highlights the significant role of the anion in the biological properties of metal complexes. The specific role of the anion in this context is to be investigated. This work has demonstrated that zinc(II) chloride complexes of imidazoles with unsaturated substituents exhibit a variety of biological activities, including experimentally observed antitumor and antifungal effects, as well as predicted antiatherosclerotic and antieczematous properties. Therefore, these complexes represent promising lead structures for further optimization and drug development.

## Figures and Tables

**Table 1:** The thermochemical parameters of the formation of the complexes from the corresponding ligands and metal salts in aqueous solution.

#	Ligand	Complex	ΔH_f_, kcal/mol	ΔG_f_, kcal/mol	logK_f_
1	*N*-Allylimidazole	**1**	−29.02	−9.63	7.06
2	*N*-Allyl-2-Methylimidazole	**2**	−33.80	−10.79	7.92
3	*N*-Propargylimidazole	**3**	−28.36	−7.63	5.59
4	*N*-Vinylimidazole	**4**	−26.25	−5.84	4.29

**Table 2:** The thermochemical parameters of the partial hydrolytic dissociation of anions from complexes in aqueous solution.

Starting Complex	ΔH, kcal/mol	ΔG, kcal/mol	logK
**1**	9.15	2.35	−1.72
**2**	10.64	1.98	−1.45
**3**	9.54	1.84	−1.35
**4**	9.61	1.77	−1.30

**Table 3:** The thermochemical parameters of the partial dissociation (hydrolysis) of ligands from complexes in aqueous solution.

Starting Complex	ΔH, kcal/mol	ΔG, kcal/mol	logK
**1**	21.99	10.44	−7.66
**2**	17.07	8.78	−6.44
**3**	17.00	7.80	−5.72
**4**	17.64	8.41	−6.17

**Table 4:** The electronic parameters of the ligands and their Zn complexes.

#	Compound	I	A	∆E	χ	η	σ	µ	S	ω	∆N_max_	*p*
1	*N*-Allylimidazole	7.48	−0.37	7.85	3.55	3.93	0.13	−3.55	0.25	1.61	0.90	5.5
2	*N*-Allyl-2-Methylimidazole	7.21	−0.57	7.78	3.32	3.89	0.13	−3.32	0.26	1.42	0.85	5.5
3	*N*-Propargylimidazole	7.58	−0.66	8.24	3.46	4.12	0.12	−3.46	0.24	1.45	0.84	5.0
4	*N*-Vinylimidazole	7.58	0.23	7.35	3.90	3.67	0.14	−3.90	0.27	2.07	1.06	4.4
5	**1**	7.98	−0.17	8.15	3.90	4.08	0.12	−3.90	0.25	1.87	0.96	16.2
6	**2**	7.70	−0.36	8.06	3.67	4.03	0.12	−3.67	0.25	1.67	0.91	21.2
7	**3**	8.09	−0.25	8.34	3.92	4.17	0.12	−3.92	0.24	1.84	0.94	15.6
8	**4**	8.00	0.61	7.39	4.31	3.70	0.14	−4.31	0.27	2.51	1.17	16.0

**Table 5:** The effect of complexes on the lifespan of mice with acute hypoxia ^1^.

Compound	Acute Hypoxic Model	Dose of Complexes, mg/kg		
		10	25	50
**1**	Hypoxia with hypercapnia	99 ± 5	98 ± 4	101 ± 7
	Hemic hypoxia	96 ± 8	103 ± 9	104 ± 11
	Hystotoxic hypoxia	97 ± 9	99 ± 5	95 ± 6
**2**	Hypoxia with hypercapnia	123 ± 4 ^a^	121 ± 4 ^b^	107 ± 8
	Hemic hypoxia	132 ± 5 ^c^	125 ± 3 ^d^	117 ± 9
	Hystotoxic hypoxia	98 ± 10	101 ± 9	99 ± 7
**3**	Hypoxia with hypercapnia	108 ± 10	120 ± 5 ^e^	119 ± 8
	Hemic hypoxia	98 ± 7	101 ± 12	96 ± 8
	Hystotoxic hypoxia	106 ± 5	102 ± 6	99 ± 6
**4**	Hypoxia with hypercapnia	106 ± 6	104 ± 6	98 ± 8
	Hemic hypoxia	102 ± 5	104 ± 10	99 ± 9
	Hystotoxic hypoxia	97 ± 6	96 ± 7	101 ± 9

^1^ Data are presented as lifespan of mice relative to control taken as 100% (*n* = 10 mice in each group): M ± m (M: arithmetic mean relative to control = 100%; m: standard error of the mean in % of M). *p*-values vs. control: ^a^ *p* = 0.026; ^b^ *p* = 0.028; ^c^ *p* = 0.011; ^d^ *p* = 0.022; ^e^ *p* = 0.037.

**Table 6:** Median cytotoxicity concentration (CC50) of the studied compounds in selected cell lines (µM, M ± SD) ^1^.

**Compound**	**Cell Lines**						**Color Legend**	
	**Vero**	**A549**	**Hep2c**	**RD**	**MT-4**	**Jurkat**	>200	max
**1**	>200	>200	131 ± 33	69 ± 8	>100	>200	200	
**2**	>200	>200	180 ± 5	70 ± 17	>100	>200	100	
**3**	>200	>200	151 ± 13	120 ± 10	>100	>200	75	
**4**	>200	>200	197 ± 4	83 ± 29	>100	150 ± 71	50	min

^1^ The compounds showing the highest antitumor activity for these cell lines are highlighted in red, while the least toxic ones are highlighted in green.

**Table 7:** Predicted activities (Pa) for the most probable biological activities of Zn complexes with substituted imidazole ligands.

Compound	Atherosclerosic	Antieczematic	Chloride Peroxidase Inhibition	Antineoplastic	Cl^−^-Transporting ATPase Inhibition	Fatty-acyl-CoA Synthase Inhibition	Phthalate 4,5-Dioxygenase Inhibition	Glucan Endo-1,6-beta-glucosidase Inhibition	Aspulvinone Dimethylallyltransferase Inhibition	27-Hydroxycholesterol 7alpha-monooxygenase Inhibition	Antibacterial	Antifungal
**1**	0.88	0.84	0.83	0.82	0.79	0.76	0.74	0.73	0.71	0.76	0.23	0.35
**2**	0.83	0.80	0.70	0.77	0.63	0.77	0.58	0.53	0.67	0.56	0.25	0.28
**3**	0.82	0.70	0.73	0.87	0.62	0.49	0.74	0.73	0.55	0.76	0.16	-
**4**	0.83	0.77	0.86	0.71	0.79	0.65	0.78	0.78	0.80	0.70	0.24	0.27

**Table 8:** Predicted physicochemical and pharmacokinetics parameters.

Complex	MR ^a^	TPSA ^b^	Log P ^c^	Log S ^d^	BBB Permeant ^e^	CYP2C19 Inhibitor	log Kp (cm/s) ^f^
**1**	76.96	26.64	1.28	−3.8	Yes	Yes	−5.93
**2**	86.9	26.64	1.87	−4.6	Yes	Yes	−5.53
**3**	74.24	26.64	0.82	−2.7	Yes	Yes	−6.66
**4**	68.93	26.64	0.89	−3.5	Yes	Yes	−5.98

^a^ MR—molar refractivity; ^b^ TPSA—topological polar surface area [70]; ^c^ LogP—lipophilicity; ^d^ LogS—solubility; ^e^ BBB—blood–brain barrier; ^f^ LogKp—skin permeation: QSPR model [71].

## Data Availability

The original contributions presented in this study are included in the article and Supplementary Material. Further inquiries can be directed to the corresponding authors.

## Supplementary Materials

The following supporting information can be downloaded at: https://www.mdpi.com/article/10.3390/ph19060874/s1, Table S1: Crystal and structure refinement data for complexes **1** and **3**; Table S2: Fractional atomic coordinates and equivalent isotropic displacement parameters for the complex **1**; Table S3: Anisotropic displacement parameters for the complex **1**; Table S4: Bond lengths for the complex **1**; Table S5: Bond angles for the complex **1**; Table S6: Hydrogen bonds for the complex **1**; Table S7: Torsion angles for the complex **1**; Table S8: Hydrogen atom coordinates and isotropic displacement parameters for the complex **1**; Table S9: Fractional atomic coordinates and equivalent isotropic displacement parameters for the complex **3**; Table S10: Anisotropic displacement parameters for the complex **3**; Table S11: Bond lengths for the complex **3**; Table S12: Bond angles for the complex **3**; Table S13: Hydrogen bonds for the complex **3**; Table S14: Torsion angles for the complex **3**; Table S15: Hydrogen atom coordinates and isotropic displacement parameters for the complex **3**; Table S16: The chemical shifts of protons in NMR spectra of compounds in different solvents; Table S17: Coordination bonds lengths; Table S18: Basic thermochemical parameters of the complexes; Table S19: Basic chemical and biological parameters of the complexes; Table S20: Correlation coefficients between the toxicity of complexes and their electronic parameters; Table S21: Cartesian coordinates of **1**; Table S22: Cartesian coordinates of **2**; Table S23: Cartesian coordinates of **3**; Table S24: Cartesian coordinates of **4**; Figure S1: Crystal packing of **1**; Figure S2: Crystal packing of **3**; Figure S3: Experimentally obtained and simulated powder X-ray diffraction diagrams for **1**; Figure S4: Experimentally obtained and simulated powder X-ray diffraction diagrams for **3**; Figure S5: ^1^H NMR spectrum of **1** in CDCl_3_; Figure S6: ^13^C NMR spectrum of **1** in CDCl_3_; Figure S7: ^1^H NMR spectrum of **2** in CDCl_3_; Figure S8: ^13^C NMR spectrum of **2** in D_2_O; Figure S9: ^1^H NMR spectrum of **3** in D_2_O; Figure S10: ^13^C NMR spectrum of **3** in D_2_O; Figure S11: ^1^H NMR spectrum of **4** in D_2_O; Figure S12: ^13^C NMR spectrum of **4** in D_2_O; Figure S13: Microphotographs of planar skin wounds in rats. Control group (intact); Figure S14: Microphotographs of planar skin wounds in rats. Exposed to Na-CMC; Figure S15: Microphotographs of planar skin wounds in rats. Exposed to ZnSO4; Figure S16: Microphotographs of planar skin wounds in rats. Exposed to **2**; Figure S17: Microphotographs of planar skin wounds in rats. Exposed to **3**; Figure S18: Microphotographs of planar skin wounds in rats. Exposed to **4**; Figure S19: Geometry of **1** optimized by the DFT; Figure S20: Geometry of **2** optimized by the DFT; Figure S21: Geometry of **3** optimized by the DFT; Figure S22: Geometry of **4** optimized by the DFT; Figure S23: Frontier molecular orbitals plot for the complex **2**; Figure S24: Frontier molecular orbitals plot for the complex **3**; Figure S25: Frontier molecular orbitals plot for the complex **4**.

## Abbreviations

The following abbreviations are used in this manuscript:

DNA	Deoxyriboucleic acid
DFT	Density functional theory
QSAR	Quantitative structure–activity relationship
NMR	Nuclear magnetic resonance
IR	Infrared
DMSO	Dimethyl sulfoxide
