# Supplementary material for: Complexes of Zinc(II) Chloride with N-Vinyl-, N-Allyl- and N-Propargylimidazoles: Structural, Theoretical and Biological Studies
Source: Pharmaceuticals (Basel). 2026 May 31;19(6):874. doi: 10.3390/ph19060874 (PMC13305716; doi:10.3390/ph19060874)
Supplement: Supplementary file 1 [file pharmaceuticals-19-00874-s001.zip › pharmaceuticals-4308520-supplementary.pdf]

Supplementary Information

Complexes of Zinc(II) Chloride with *N*-Vinyl-, *N*-Allyl- and *N*-Propargylimidazoles: Structural, Theoretical and Biological Studies

Vladimir S. Tyurin [1,*], Victoria S. Babasieva [2], Mikhail S. Grigoriev [1], Lidiya N. Parshina [3], Ilya A. Zamilatskov [2], Elena A. Smolyarchuk [2], Olga V. Nesterova [2], Vladislav N. Turenko [2], Tatiana I. Kolyganova [2,4], Vera G. Arzumanian [4], Kerim Mutig [5], Mikhail Yu. Samsonov [6] and Svetlana A. Lebedeva [2,5,*]

[1] Frumkin Institute of Physical Chemistry and Electrochemistry, Russian Academy of Sciences, 119071 Moscow, Russia; grigoriev@ipc.rssi.ru

[2] The Institute of Pharmacy, Sechenov First Moscow State Medical University (Sechenov University), 119048 Moscow, Russia; victoriababasieva@mail.ru (V.S.B.); joz@mail.ru (I.A.Z.); smolyarchuk_e_a@staff.sechenov.ru (E.A.S.); nesterova_o_v@staff.sechenov.ru (O.V.N.); turenko_v_n@staff.sechenov.ru (V.N.T.); kolyganova_t_i@staff.sechenov.ru (T.I.K.)

[3] A. E. Favorsky Irkutsk Institute of Chemistry, Siberian Branch of the Russian Academy of Sciences, 664033 Irkutsk, Russia; parshina@irioch.irk.ru

[4] Mechnikov Research Institute for Vaccines and Sera, 105064 Moscow, Russia; veraar@mail.ru

[5] Scientific Center of Genetics and Life Sciences, Sirius University of Science and Technology, 354340 Sirius, Russia; mutig.k@talantiusheh.ru

[6] Medical Department, R-Pharm JSC, 123154 Moscow, Russia; samsonov@rpharm.ru

* Correspondence: tv@org.chem.msu.ru (V.S.T.); lebedeva502@yandex.ru (S.A.L.)

Contents

1. Synthesis 2
2. X-Ray data 4
3. NMR spectra 13
4. Microphotographs 18
5. DFT calculations data 21
6. References 28

Synthesis

General

NMR spectra were recorded using a Bruker Avance III 600 spectrometer (600.13 or 150.90 MHz for 1H and 13C, respectively, in $CDCl_3$, DMSO-d6 and D_2O (99%). All measurements were carried out at 303 K. FTIR spectra for complexes were run on a Bruker Vertex 70 instrument Melting points (uncorrected) were measured on a Kofler micro hot-stage apparatus. Elemental analyses for C, H, N were performed on a Flash EA 1112 CHNS-O/MAS analyzer. The Cl content was determined by a method involving mineralization of samples according to Sheniger with subsequent titration of chlorides by Hg(II). N-Allylimidazole, N-Vinylimidazole, N-Propargylimidazole and allyl bromide were purified by vacuum distillation. All the other chemicals were purchased from commercial suppliers and used without further purification. Solvents were purified according to a standard protocol.

N-Allyl-2-methylimidazole was prepared from 2-methylimidazole and allyl bromide according to the described procedure [1] with minor modifications. Solvents were dried and distilled according to the standard methods. Complexes **3** [2] and **4** [3] were prepared according to the reported procedures.

N-allyl-2-methylimidazole

2-Methylimidazole (8.20 g, 100 mmol) and NaOH (4.2 g, 105 mmol) in DMSO (50 mL) were stirred for 30 min. Allyl bromide (6.33 г, 52.3 mmol) was added at 10-15 °C for 30 min. The process was accompanied by a strong thickening of the reaction mixture. The next day, 50 ml of water was added to the reaction mixture, and the crude product was extracted with $CHCl_3$ three times. The combined organic phase was washed with distilled water and dried over anhydrous $MgSO_4$, and the solvent was removed under reduced pressure. The residue was purified by distillation in vacuo to give pure N-allyl-2-methylimidazole as colorless liquid; yield: 5.53 g (45%); bp 68-70 °C (2 mm Hg). Anal. Calc. for $C_7H_{10}N_2$: C, 68.82; H, 8.25; N, 22.93. Found: C, 68.42; H, 7.96; N, 22.98%. ^{1}H NMR, DMSO-d6, δ (ppm): 6.89 (s, 1H, C^4H), 6.79 (s, 1H, C^5H), 5.88 (m, 1H, $CH_2C\underline{H}$=C), 5.20 (d, 1H, J = 10.2 Hz, $CH=C\underline{H}_{cis}$), 4.96 (d, 1H, J = 17.1 Hz, $CH=C\underline{H}_{trans}$), 4.43 (d, 2H, J = 4.6 Hz, NCH_2), 2.32 (s, 3H, CH_3).

Dichlorobis(*N*-allylimidazole)zinc(II) (1)

$ZnCl_2$ (0.63 g, 4.6 mmol) was added portion-wise to a stirred solution of N-allylimidazole (1.20 g, 11.1 mmol) in ether-ethanol (3:1) mixture (40 mL). The suspension was stirred at ambient temperature for 18 h. The white precipitate formed was filtered, washed with ether and dried under vacuum. Yield: 1.46 g (90%), m.p. 130–131 °C. Anal. Calc. for $C_{12}H_{16}Cl_2N_4Zn$: C, 40.88; H, 4.57; Cl, 20.11; N, 15.89. Found: C, 40.65; H, 4.46; Cl, 20.14; N, 15.45%. IR (selected bands, KBr, cm^{-1}): 1647 ($C=C_{allyl}$), 1526 ($C=C_{imidazole}$, C=N). ^{1}H NMR, $CDCl_3$, δ (ppm): 8.02 (br. s, 1H, C^2H), 7.21 (br. s, 1H, C^5H), 7.01 (t, 1H, J = 1.5 Hz, C^4H), 5.97 (m, 1H, $CH_2C\underline{H}$=C), 5.40 (d, 1H, J = 10.2 Hz, $CH=C\underline{H}_{cis}$), 5.32 (d, 1H, J = 17.0 Hz, $CH=C\underline{H}_{trans}$), 4.62 (d, 2H, J = 5.5 Hz, NCH_2). ^{1}H NMR, D_2O, δ (ppm): 8.02 (t, 1H, J = 1.3 Hz, C^2H), 7.29 (t, 1H, J = 1.5 Hz, C^5H), 7.14 (t, 1H, J = 1.4 Hz, C^4H), 6.02 (m, 1H, $CH_2C\underline{H}$=C), 5.31 (d, 1H, J = 10.3 Hz, $CH=C\underline{H}_{cis}$), 5.19 (d, 1H, J = 17.1 Hz, $CH=C\underline{H}_{trans}$), 4.71 (m, 2H, m, NCH_2). ^{1}H NMR, DMSO-d6, δ (ppm): 8.08 (s, 1H, C^2H), 7.36 (s, 1H, C^5H), 7.06 (s, 1H, C^4H), 6.02 (m, 1H, $CH_2C\underline{H}$=C), 5.25 (dd, 1H, J = 10.3 Hz, J = 1.5 Hz, $CH=\underline{CH}_{cis}$), 5.16 (dd, 1H, J = 17.0 Hz, J = 1.5 Hz, $CH=C\underline{H}_{trans}$), 4.74 (d, 2H, J = 6.0 Hz, NCH_2). ^{13}C NMR, $CDCl_3$, δ (ppm): 138.0 (C^2H), 131.1 ($\underline{C}H=CH_2$), 127.6 (C^5H), 120.6 (C^4H), 120.0 ($CH=\underline{C}H_2$), 50.5 (NCH_2). ^{13}C NMR, D_2O, δ (ppm): 138.0 (C^2H), 132.3 ($\underline{C}H=CH_2$), 126.0 (C^5H), 121.3 (C^4H), 119.0 ($CH=\underline{C}H_2$), 50.0 (NCH_2). ^{13}C NMR, DMSO-d6, δ (ppm): 139.1 (C^2H), 134.4 ($\underline{C}H=CH_2$), 128.1 (C^5H), 120.8 (C^4H), 118.5 ($CH=\underline{C}H_2$), 50.4 (NCH_2).

Dichlorobis(*N*-allyl-2-methylimidazole)zinc(II) (2)

Complex **2** was prepared by the same procedure as complex **1** from $ZnCl_2$ (0.56 g, 4.1 mmol) and N-allyl-2-methylimidazole (1.20 g, 9.8 mmol) in ether-ethanol (3:1) mixture (25 mL) for 12 h, yielding 1.32 g (85%) of **2** as colorless powder, m.p. 125-127 °C. Anal. Calcd for $C_{14}H_{20}Cl_2N_4Zn$: C, 44.18; H, 5.30; Cl, 18.63; N, 14.72. Found: C, 44.32; H, 5.21; Cl, 18.80; N, 14.63. IR (KBr, cm^{-1}): 1642 ($C=C_{allyl}$), 1542, ($C=C_{imidazole}$, $C=N_{imidazole}$). ^1H NMR, $CDCl_3$, δ (ppm): 7.13 (d, 1H, J = 1.5 Hz, C^5H), 6.88 (d, 1H, J = 1.5 Hz, C^4H), 5.91 (m, 1H, $CH_2C\underline{H}=C$), 5.34 (d, 1H, J = 10.3 Hz, $CH=C\underline{H}_{cis}$), 5.11 (d, 1H, J = 17.1 Hz, $CH=C\underline{H}_{trans}$), 4.52 (d, 2H, J = 5.5 Hz, NCH_2), 2.61 (s, 3H, CH_3). ^1H NMR, D_2O, δ (ppm): 7.23 (d, 1H, J = 1.9 Hz, C^5H), 7.09 (d, 1H, J = 1.9 Hz, C^4H), 5.98 (m, 1H, $CH_2C\underline{H}=C$), 5.30 (d, 1H, J = 10.4 Hz, $CH=C\underline{H}_{cis}$), 5.04 (d, 1H, J = 17.2 Hz, $CH=C\underline{H}_{trans}$), 4.66 (dt, 2H, J_1 = 5.3 Hz, J_2 = 1.7 Hz, NCH_2), 2.44 (s, 3H, CH_3). ^1H NMR, DMSO-d6, δ (ppm): 7.27 (s, 1H, C^5H), 6.93 (s, 1H, C^4H), 5.97 (m, 1H, $CH_2C\underline{H}=C$), 5.22 (d, 1H, J = 10.3 Hz, $CH=C\underline{H}_{cis}$), 4.96 (d, 1H, J = 18.2 Hz, $CH=C\underline{H}_{trans}$), 4.64 (d, 2H, J = 5.3 Hz, NCH_2), 2.38 (s, 3H, CH_3). ^{13}C NMR, D_2O, δ (ppm): 145.64 (C^2), 131.39 ($\underline{C}H=CH_2$), 121.65 (C^5), 121.37 (C^4), 118.26 ($CH=\underline{C}H_2$), 49.03 (N-CH_2), 10.71 (CH_3).^{13}C NMR, DMSO-d6, δ (ppm): 145.88 (C^2), 132.51 ($\underline{C}H=CH_2$), 125.41 (C^5), 121.63 (C^4), 117.54 ($CH=\underline{C}H_2$), 47.30 (N-CH_2), 11.73 (CH_3).

Dichlorobis(*N*-propargylimidazole)zinc(II) (3)

$ZnCl_2$ (0.4 g, 2.9 mmol) was added portion-wise to a stirred solution of N-propargylimidazole (0.7 g, 6.6 mmol) in EtOH (6 mL) The suspension was stirred at ambient temperature for 24 h. A formed precipitate was filtered off, sequentially washed with ether, and then dried under vacuum. Yield: 0.95 g (93%), m.p. 135–137 °C. ^1H NMR, $CDCl_3$, δ (ppm): 8.15 (br. s, 1H, C^2H), 7.25 (br. s, 1H, C^5H), 7.16 (br. s, 1H, C^4H), 4.82 (br.s, 2H, NCH_2), 2.61 (t, 1H, J = 2.2 Hz, C≡CH). ^1H NMR, D_2O, δ (ppm): 8.02 (br. s, 1H, C^2H), 7.37 (br. s, 1H, C^5H), 7.12 (br. s, 1H, C^4H), 4.93 (d, 2H, J = 2.6 Hz, NCH_2), 2.92 (t, 1H, J = 2.6 Hz, C≡CH). ^1H NMR, DMSO-d6, δ (ppm): 8.06 (br. s, 1H, C^2H), 7.42 (br. s, 1H, C^5H), 7.05 (br. s, 1H, C^4H), 5.02 (d, 2H, J = 2.5 Hz, NCH_2), 3.60 (t, 1H, J = 2.5 Hz, C≡CH). ^{13}C NMR, D_2O, δ (ppm): 137.95 (C^2), 126.87 (C^5), 120.70 (C^4), 76.75 ($\underline{C}$≡CH), 75.62 (C≡$\underline{C}$H), 37.02 (N-CH_2). ^{13}C NMR, DMSO-d6, δ (ppm): 141.10 (C^2), 132.34 (C^5), 125.07 (C^4), 82.61 ($\underline{C}$≡CH), 79.62 (C≡$\underline{C}$H), 39.32 (N-CH_2).

Dichlorobis(*N*-vinylimidazolyl)zinc(II) (4)

$ZnCl_2$ (0.72 g, 5.3 mmol) was added portion-wise to a stirred solution of N-vinylimidazole (1.0 g, 10.6 mmol) in acetone (15 mL). When stirring for 3 h, the light suspension gradually gave a clear solution. The solution was evaporated to 1/3 of the initial volume. Then, ether (50 mL) was added to the solution. The white precipitate formed was filtered, washed with ether and dried under vacuum. Yield: 1.47 g (85%), m.p. 134–135 °C. ^1H NMR, $CDCl_3$, δ (ppm): 8.21 (br.s, 1H, C^2H), 7.28 (br.m, 2H, C^4H, C^5H), 6.95 (dd, 1H, J_1 = 15.7 Hz, J_2 = 8.8 Hz, $C\underline{H}=CH_2$), 5.50 (dd, 1H, J_1 = 15.7 Hz, J_2 = 2.3 Hz, $CH=C\underline{H}_{trans}$), 5.18 (dd, 1H, J_1 = 8.8 Hz, J_2 = 2.3 Hz, $CH=C\underline{H}_{cis}$). ^1H NMR, D_2O, δ (ppm): 8.09 (t, 1H, J = 1.2 Hz, C^2H), 7.54 (t, 1H, J = 1.4 Hz, C^5H), 7.14 (t, 1H, J = 1.2 Hz, C^4H), 7.09 (dd, 1H, J_1 = 15.7 Hz, J_2 = 8.8 Hz, $C\underline{H}=CH_2$), 5.55 (dd, 1H, J_1 = 15.7 Hz, J_2 = 2.1 Hz, $CH=C\underline{H}_{trans}$), 5.12 (dd, 1H, J_1 = 8.8 Hz, J_2 = 2.1 Hz, $CH=C\underline{H}_{cis}$). ^1H NMR, DMSO-d6, δ (ppm): 8.36 (br.s, 1H, C^2H), 7.87 (br.s, 1H, C^5H), 7.28 (dd, 1H, J_1 = 16 Hz, J_2 = 9 Hz, $C\underline{H}=CH_2$), 6.95 (br.s, 1H, C^4H), 5.70 (dd, 1H, J_1 = 16 Hz, J_2 = 2 Hz, $CH=C\underline{H}_{trans}$), 5.07 (dd, 1H, J_1 = 9 Hz, J_2 = 2 Hz, $CH=C\underline{H}_{cis}$). ^{13}C NMR, $CDCl_3$, δ (ppm): 137.08 (C^2), 128.67 ($\underline{C}H=CH_2$), 128.19 (C^5), 117.06 (C^4), 105.88 ($CH=\underline{C}H_2$).^{13}C NMR, D_2O, δ (ppm): 137.35 (C^2), 129.06 ($\underline{C}H=CH_2$), 127.46 (C^5), 117.62 (C^4), 104.85 ($CH=\underline{C}H_2$). ^3C NMR, DMSO-d6, δ (ppm): 137.57 (C^2), 129.36 ($\underline{C}H=CH_2$), 127.30 (C^5), 119.14 (C^4), 105.12 ($CH=\underline{C}H_2$).

X-ray data

Experimental

Single crystals of dichlorobis(*N*-allylimidazole)zinc(II) (**1**) and dichlorobis(*N*-allyl-2-methylimidazole)zinc(II) (**3**) were obtained by slow diffusion of diethyl ether into a acetone solution. X-ray single crystal diffraction analysis was performed with a Bruker KAPPA APEX II area-detector diffractometer. The crystals were kept at 100(2) K during data collection The experimental intensities were corrected for absorption using the SADABS program [4]. The structures were solved by the intrinsic phasing method in SHELXT [5] and refined by the full-matrix least-squares method SHELXL [6] on F^2 for all data in the anisotropic approximation for all non-H atoms. Hydrogen atoms were placed geometrically and refined using a riding model with U_{iso} constrained at 1.2–1.5 times U_{eq} of the carrier C or N atoms atoms, except for the terminal CH groups of the propargyl fragments. These H atoms were located from a difference Fourier map and refined without any limitations.

The crystal data, data collection and refinement parameters are given in Table S1. Crystallographic data for have been deposited with the Cambridge Crystallographic Data Centre, depositions CCDC 2547382-2547383.

Table S1. Crystal and structure refinement data for complexes **1** and **3**.

Identification code	ALL-Cl (**1**)	Propargyl-Cl (**3**)
Empirical formula	$C_{12}H_{16}Cl_2N_4Zn$	$C_{12}H_{12}Cl_2N_4Zn$
Formula weight	352.56	348.53
Temperature, K	100(2)	100(2)
Crystal system	orthorhombic	orthorhombic
Space group	Pbca	Pbca
a, Å	8.1414(6)	8.0702(5)
b, Å	15.3349(12)	15.3964(10)
c, Å	24.8985(19)	23.9338(14)
α, °	90	90
β, °	90	90
γ, °	90	90
Volume, Å^3	3108.5(4)	2973.8(3)
Z	8	8
ρ_{calc}, g/cm^3	1.507	1.557
μ, mm^{-1}	1.915	2.001
F(000)	1440.0	1408
Crystal size, mm	0.42 × 0.36 × 0.32	0.4 × 0.38 × 0.34
Radiation	MoKα (λ = 0.71073)	MoKα (λ = 0.71073)
2Θ range for data collection, °	8.242 to 59.996	8.48 to 59.996
Index ranges	$-11 \le h \le 11$, $-18 \le k \le 21$, $-34 \le l \le 35$	$-11 \le h \le 11$, $-21 \le k \le 21$, $-33 \le l \le 32$
Reflections collected	52298	55092
Independent reflections	4524 [R_{int} = 0.0502, R_{sigma} = 0.0261]	4327 [R_{int} = 0.0606, R_{sigma} = 0.0299]
Data/restraints/parameters	4524/0/172	4327/0/180
Goodness-of-fit on F^2	1.055	1.029
Final R indexes [I>=2σ (I)]	R_1 = 0.0259, wR_2 = 0.0526	R_1 = 0.0276, wR_2 = 0.0537
Final R indexes [all data]	R_1 = 0.0374, wR_2 = 0.0559	R_1 = 0.0437, wR_2 = 0.0588
Largest diff. peak/hole , e Å^{-3}	0.43/-0.31	0.44/-0.33

Table S2. Fractional atomic coordinates (×10^4) and equivalent isotropic displacement parameters (Å^2×10^3) for the complex **1**.

**Atom**	***x***	***y***	***z***	***U(eq)***[1]
Zn1	5609.0(2)	4943.0(2)	3551.1(2)	12.08(5)
Cl1	6803.8(5)	5471.6(2)	4295.5(2)	19.69(8)
Cl2	7019.4(5)	3974.6(2)	3049.0(2)	15.62(8)
N11	1281.7(16)	3998.6(8)	4218.7(5)	14.3(2)
N13	3462.0(15)	4409.1(8)	3764.3(5)	14.6(3)
N21	4796.5(16)	6611.0(8)	2267.0(5)	14.5(3)
N23	5200.5(16)	5920.7(8)	3030.1(5)	14.2(2)
C12	2850.3(18)	4267.7(9)	4250.7(6)	14.4(3)
C14	2216.7(19)	4219.9(10)	3406.8(6)	17.0(3)
C15	860.3(19)	3969.4(10)	3683.6(6)	16.9(3)
C16	153.7(19)	3815.8(10)	4666.8(6)	16.7(3)
C17	-724(2)	2966.7(10)	4583.7(6)	18.8(3)
C18	-2317(2)	2912.7(11)	4498.9(7)	25.5(4)
C22	4862.1(19)	5832.4(9)	2513.1(6)	16.0(3)
C24	5363.5(19)	6805.1(9)	3116.1(6)	17.0(3)
C25	5120(2)	7234.6(10)	2647.2(6)	18.7(3)
C26	4550(2)	6760.8(10)	1689.2(6)	17.3(3)
C27	6150(2)	6865.3(11)	1400.7(7)	22.8(3)
C28	6595(2)	6371.3(12)	995.6(7)	27.8(4)

[1] U_{eq} is defined as 1/3 of the trace of the orthogonalised U_{IJ} tensor.

Table S3. Anisotropic displacement parameters (Å^2×10^3) for the complex **1**. The Anisotropic displacement factor exponent takes the form: $-2\pi^2[h^2a^{*2}U_{11}+2hka^*b^*U_{12}+\ldots]$.

**Atom**	U_{11}	U_{22}	U_{33}	U_{23}	U_{13}	U_{12}
Zn1	13.37(8)	12.86(8)	10.00(8)	0.26(6)	-0.38(7)	-1.44(6)
Cl1	24.25(19)	22.52(18)	12.32(17)	-1.87(14)	-4.02(15)	-5.21(15)
Cl2	16.49(17)	16.42(16)	13.96(17)	-2.54(13)	-0.78(13)	1.74(13)
N11	13.3(6)	17.0(6)	12.4(6)	1.7(5)	-1.4(5)	-1.8(5)
N13	14.2(6)	16.3(6)	13.5(6)	0.3(5)	-1.0(5)	-1.9(5)
N21	16.1(6)	15.7(6)	11.8(6)	-0.1(5)	-2.8(5)	0.8(5)
N23	16.5(6)	13.8(6)	12.4(6)	0.3(5)	-0.5(5)	0.4(5)
C12	14.1(7)	17.3(7)	12.0(7)	-0.1(5)	-2.4(6)	-1.4(5)
C14	20.4(8)	19.0(7)	11.6(7)	-0.4(5)	-3.3(6)	-2.8(6)
C15	16.2(7)	21.7(7)	12.9(7)	0.2(6)	-5.0(6)	-1.9(6)
C16	15.5(7)	21.5(7)	13.0(7)	-0.5(6)	1.4(6)	-2.7(6)
C17	24.2(8)	18.0(7)	14.3(7)	2.5(6)	3.5(6)	-2.4(6)
C18	27.1(9)	25.4(8)	23.9(9)	1.0(7)	2.5(7)	-10.4(7)
C22	19.8(8)	13.6(6)	14.6(7)	-1.0(6)	-1.1(6)	-1.2(6)
C24	21.1(8)	14.8(7)	15.3(7)	-2.4(6)	-3.8(6)	0.4(6)
C25	25.5(8)	13.4(7)	17.3(8)	-1.8(6)	-4.1(7)	-0.6(6)
C26	21.2(8)	19.9(7)	10.7(7)	1.3(6)	-3.8(6)	2.3(6)
C27	24.8(9)	23.8(8)	19.9(8)	4.6(6)	-1.7(7)	-5.0(7)
C28	28.5(10)	33.8(9)	21.1(9)	4.2(7)	1.3(7)	3.9(8)

Table S4. Bond lengths for the complex **1**.

Atom	Atom	Length/Å	Atom	Atom	Length/Å
Zn1	Cl1	2.2447(4)	N21	C25	1.3711(19)
Zn1	Cl2	2.2555(4)	N21	C26	1.4706(19)
Zn1	N13	2.0020(13)	N23	C22	1.3232(19)
Zn1	N23	2.0103(12)	N23	C24	1.3795(18)
N11	C12	1.3445(19)	C14	C15	1.357(2)
N11	C15	1.3767(19)	C16	C17	1.500(2)
N11	C16	1.4720(19)	C17	C18	1.316(2)
N13	C12	1.3273(19)	C24	C25	1.355(2)
N13	C14	1.3800(19)	C26	C27	1.496(2)
N21	C22	1.3431(18)	C27	C28	1.313(2)

Table S5. Bond angles for the complex **1**.

Atom	Atom	Atom	Angle/°	Atom	Atom	Atom	Angle/°
Cl1	Zn1	Cl2	118.348(16)	C25	N21	C26	126.34(12)
N13	Zn1	Cl1	107.88(4)	C22	N23	Zn1	125.86(10)
N13	Zn1	Cl2	108.80(4)	C22	N23	C24	105.77(12)
N13	Zn1	N23	109.37(5)	C24	N23	Zn1	128.10(10)
N23	Zn1	Cl1	109.59(4)	N13	C12	N11	110.63(13)
N23	Zn1	Cl2	102.57(4)	C15	C14	N13	109.25(13)
C12	N11	C15	107.70(13)	C14	C15	N11	106.23(13)
C12	N11	C16	127.31(13)	N11	C16	C17	110.99(12)
C15	N11	C16	124.89(13)	C18	C17	C16	123.11(16)
C12	N13	Zn1	129.52(10)	N23	C22	N21	111.16(13)
C12	N13	C14	106.18(12)	C25	C24	N23	109.26(13)
C14	N13	Zn1	123.80(10)	C24	C25	N21	106.49(13)
C22	N21	C25	107.31(12)	N21	C26	C27	111.56(13)
C22	N21	C26	126.20(12)	C28	C27	C26	123.21(16)

Table S6. Hydrogen bonds for the complex **1**.

D	H	A	d(D-H)/Å	d(H-A)/Å	d(D-A)/Å	D-H-A/°
C12	H12	$Cl1^{1}$	0.95	2.83	3.6524(16)	145.6
C14	H14	$Cl2^{2}$	0.95	2.72	3.6476(16)	164.5
C15	H15	$Cl2^{3}$	0.95	2.60	3.5035(16)	158.6
C26	H26A	$Cl2^{4}$	0.99	2.79	3.6853(16)	150.4

^{1}1-X,1-Y,1-Z; ^{2}-1/2+X,+Y,1/2-Z; ^{3}-1+X,+Y,+Z; ^{4}1-X,1/2+Y,1/2-Z

Table S7. Torsion angles for the complex **1**.

A	B	C	D	Angle/°	A	B	C	D	Angle/°
Zn1	N13	C12	N11	172.03(10)	C15	N11	C12	N13	-0.28(17)
Zn1	N13	C14	C15	-172.32(10)	C15	N11	C16	C17	50.67(19)
Zn1	N23	C22	N21	-174.63(10)	C16	N11	C12	N13	-176.78(13)
Zn1	N23	C24	C25	174.24(11)	C16	N11	C15	C14	177.05(13)
N11	C16	C17	C18	-113.29(18)	C22	N21	C25	C24	-0.35(18)
N13	C14	C15	N11	-0.45(17)	C22	N21	C26	C27	-94.79(18)
N21	C26	C27	C28	122.89(17)	C22	N23	C24	C25	-0.04(18)

A	B	C	D	Angle/°	A	B	C	D	Angle/°
N23	C24	C25	N21	0.24(19)	C24	N23	C22	N21	-0.19(18)
C12	N11	C15	C14	0.45(17)	C25	N21	C22	N23	0.34(18)
C12	N11	C16	C17	-133.40(15)	C25	N21	C26	C27	80.13(19)
C12	N13	C14	C15	0.28(17)	C26	N21	C22	N23	176.06(14)
C14	N13	C12	N11	0.00(16)	C26	N21	C25	C24	-176.06(14)

Table S8. Hydrogen atom coordinates (Å×10^4) and isotropic displacement parameters (Å^2×10^3) for the complex **1**.

Atom	*x*	*y*	*z*	*U(eq)*
H12	3439.63	4345.75	4576.53	17
H14	2296.4	4258.85	3026.86	20
H15	-172.83	3806.45	3537.31	20
H16A	-660.38	4293.02	4696.13	20
H16B	782.08	3792.46	5006.91	20
H17	-101.65	2442.98	4592.82	23
H18A	-2965.68	3427.3	4488.06	31
H18B	-2817.47	2359.12	4448.45	31
H22	4688.36	5289.08	2338.77	19
H24	5607.78	7071.12	3451.33	20
H25	5164.85	7847.26	2592.53	22
H26A	3877.76	7292.33	1637.78	21
H26B	3940.85	6262.34	1533.59	21
H27	6878.77	7311.08	1516.42	27
H28A	5888.06	5921.05	872.37	33
H28B	7625.5	6463.93	825.63	33

Table S9. Fractional atomic coordinates (×10^4) and equivalent isotropic displacement parameters (Å^2×10^3) for the complex **3**. U_{eq} is defined as 1/3 of the trace of the orthogonalised U_{IJ} tensor**.**

Atom	*x*	*y*	*z*	*U(eq)*
Zn1	5447.6(2)	5478.0(2)	6520.1(2)	14.11(5)
Cl1	6774.3(5)	4959.6(3)	5749.9(2)	18.51(9)
Cl2	6868.3(5)	6564.0(3)	6931.3(2)	18.21(9)
N11	1252.6(18)	6163.0(9)	5642.0(5)	15.7(3)
N13	3277.1(18)	5907.0(9)	6233.1(5)	15.7(3)
N21	5216.6(18)	3852.5(9)	7892.7(5)	16.7(3)
N23	5300.9(18)	4524.1(9)	7085.3(5)	15.6(3)
C12	2811(2)	5874.4(11)	5701.9(6)	17.2(3)
C14	1936(2)	6236.6(12)	6522.4(7)	19.4(4)
C15	674(2)	6398.9(12)	6161.7(7)	20.9(4)
C16	385(2)	6273.9(12)	5105.7(7)	18.6(3)
C17	788(2)	7104.7(12)	4842.1(7)	20.0(4)
C18	1131(3)	7767.3(15)	4627.4(8)	30.8(5)
C22	4931(2)	4600.2(11)	7623.6(7)	16.7(3)
C24	5844(2)	3680.8(11)	7015.0(7)	18.2(3)
C25	5800(2)	3261.5(12)	7512.0(7)	20.2(4)
C26	4976(2)	3685.7(13)	8491.6(7)	21.3(4)

Atom	*x*	*y*	*z*	*U(eq)*
C27	6548(2)	3707.0(12)	8801.5(7)	20.4(4)
C28	7798(3)	3708.3(15)	9056.4(8)	29.8(5)

Table S10. Anisotropic displacement parameters ($Å^2 \times 10^3$) for the complex **3**. The anisotropic displacement factor exponent takes the form: $-2\pi^2[h^2a^{*2}U_{11}+2hka^*b^*U_{12}+\ldots]$.

Atom	U_{11}	U_{22}	U_{33}	U_{23}	U_{13}	U_{12}
Zn1	16.53(10)	15.28(10)	10.51(9)	1.08(7)	-1.00(7)	1.44(8)
Cl1	20.9(2)	21.3(2)	13.36(17)	-1.47(15)	1.51(15)	4.53(17)
Cl2	21.5(2)	18.0(2)	15.06(17)	-2.60(15)	-0.17(15)	-2.06(17)
N11	16.1(7)	18.8(7)	12.4(6)	2.1(5)	0.4(5)	0.8(6)
N13	17.5(7)	17.4(7)	12.4(6)	1.8(5)	0.2(5)	0.7(6)
N21	18.7(7)	18.4(8)	13.2(6)	2.5(5)	2.0(5)	2.1(6)
N23	18.3(7)	15.1(7)	13.4(6)	1.3(5)	-1.1(5)	0.8(6)
C12	17.2(9)	21.7(9)	12.7(7)	0.7(6)	1.2(6)	2.7(7)
C14	21.6(9)	23.4(9)	13.1(7)	-0.7(7)	3.0(6)	0.9(7)
C15	18.2(9)	27.0(10)	17.5(8)	-0.9(7)	5.0(6)	4.2(7)
C16	18.2(9)	23.4(9)	14.2(7)	1.3(6)	-2.5(6)	1.4(7)
C17	20.8(9)	24.2(9)	15.1(7)	-0.2(7)	-5.0(6)	0.0(7)
C18	34.0(12)	30.7(12)	27.8(10)	8.6(9)	-14.2(9)	-7.0(9)
C22	18.3(8)	16.1(8)	15.9(8)	-1.8(6)	0.5(6)	1.0(7)
C24	22.0(9)	16.0(8)	16.5(7)	-1.3(6)	3.0(6)	2.0(7)
C25	26.0(10)	16.2(8)	18.5(8)	1.3(7)	5.0(7)	4.4(7)
C26	24.5(9)	26.7(10)	12.8(7)	4.0(7)	4.7(6)	0.3(7)
C27	26.2(10)	21.8(9)	13.1(7)	2.3(7)	4.8(7)	-2.1(8)
C28	27.1(11)	40.5(13)	21.8(9)	5.3(9)	1.8(8)	-4.7(9)

Table S11. Bond lengths for the complex **3**.

Atom	Atom	Length/Å	Atom	Atom	Length/Å
Zn1	Cl1	2.2763(4)	N21	C25	1.371(2)
Zn1	Cl2	2.2537(5)	N21	C26	1.469(2)
Zn1	N13	1.9941(14)	N23	C22	1.328(2)
Zn1	N23	2.0003(14)	N23	C24	1.381(2)
N11	C12	1.342(2)	C14	C15	1.359(2)
N11	C15	1.378(2)	C16	C17	1.463(2)
N11	C16	1.472(2)	C17	C18	1.175(3)
N13	C12	1.3266(19)	C24	C25	1.354(2)
N13	C14	1.381(2)	C26	C27	1.470(3)
N21	C22	1.339(2)	C27	C28	1.178(3)

Table S12. Bond angles for the complex **3**.

Atom	Atom	Atom	Angle/°	Atom	Atom	Atom	Angle/°
Cl2	Zn1	Cl1	111.993(18)	C25	N21	C26	125.29(15)
N13	Zn1	Cl1	104.50(4)	C22	N23	Zn1	127.21(12)
N13	Zn1	Cl2	110.58(4)	C22	N23	C24	105.83(14)
N13	Zn1	N23	115.10(6)	C24	N23	Zn1	126.10(11)
N23	Zn1	Cl1	108.55(4)	N13	C12	N11	110.83(14)

Atom	Atom	Atom	Angle/°	Atom	Atom	Atom	Angle/°
N23	Zn1	Cl2	106.23(4)	C15	C14	N13	109.66(14)
C12	N11	C15	107.97(14)	C14	C15	N11	105.72(15)
C12	N11	C16	125.27(14)	C17	C16	N11	111.83(14)
C15	N11	C16	126.56(15)	C18	C17	C16	179.1(2)
C12	N13	Zn1	124.51(12)	N23	C22	N21	110.61(15)
C12	N13	C14	105.81(14)	C25	C24	N23	109.44(15)
C14	N13	Zn1	129.57(11)	C24	C25	N21	106.05(15)
C22	N21	C25	108.07(13)	N21	C26	C27	111.98(14)
C22	N21	C26	126.64(15)	C28	C27	C26	178.5(2)

Table S13. Hydrogen bonds for the complex **3**.

D	H	A	d(D-H)/Å	d(H-A)/Å	d(D-A)/Å	D-H-A/°
C12	H12	Cl1[1]	0.95	2.94	3.7195(17)	140.5
C14	H14	Cl2[2]	0.95	2.79	3.7354(17)	177.8
C15	H15	Cl2[3]	0.95	2.75	3.5901(18)	147.6
C16	H16A	Cl1[1]	0.99	2.75	3.6130(18)	145.6
C26	H26B	Cl1[2]	0.99	2.76	3.7173(19)	163.6

[1]1-X,1-Y,1-Z; [2]-1/2+X,+Y,3/2-Z; [3]-1+X,+Y,+Z

Table S14. Torsion angles for the complex **3**.

A	B	C	D	Angle/°	A	B	C	D	Angle/°
Zn1	N13	C12	N11	176.30(11)	C15	N11	C16	C17	91.0(2)
Zn1	N13	C14	C15	-176.16(13)	C16	N11	C12	N13	175.45(15)
Zn1	N23	C22	N21	-169.68(12)	C16	N11	C15	C14	-175.34(16)
Zn1	N23	C24	C25	169.75(12)	C22	N21	C25	C24	-0.1(2)
N13	C14	C15	N11	0.1(2)	C22	N21	C26	C27	-101.5(2)
N23	C24	C25	N21	0.2(2)	C22	N23	C24	C25	-0.2(2)
C12	N11	C15	C14	-0.2(2)	C24	N23	C22	N21	0.13(19)
C12	N11	C16	C17	-83.4(2)	C25	N21	C22	N23	0.0(2)
C12	N13	C14	C15	0.0(2)	C25	N21	C26	C27	77.6(2)
C14	N13	C12	N11	-0.1(2)	C26	N21	C22	N23	179.25(16)
C15	N11	C12	N13	0.2(2)	C26	N21	C25	C24	-179.39(16)

Table S15. Hydrogen atom coordinates (Å×10^4) and isotropic displacement parameters (Å^2×10^3) for the complex **3**.

Atom	*x*	*y*	*z*	*U(eq)*
H12	3489.44	5673.73	5404.28	21
H14	1902.37	6334.84	6914.11	23
H15	-387.87	6627.65	6249.71	25
H16A	697.72	5794.53	4850.9	22
H16B	-825.25	6241.14	5169.13	22
H18	1410(30)	8273(17)	4471(10)	51(8)
H22	4520.27	5114.03	7794.27	20
H24	6195.22	3433.17	6671.18	22
H25	6110.04	2676.46	7582.69	24
H26A	4451.31	3109.54	8540.81	26
H26B	4216.19	4128.6	8647.51	26

**Atom**	***x***	***y***	***z***	***U(eq)***
H28	8780(40)	3728(17)	9225(11)	56(8)

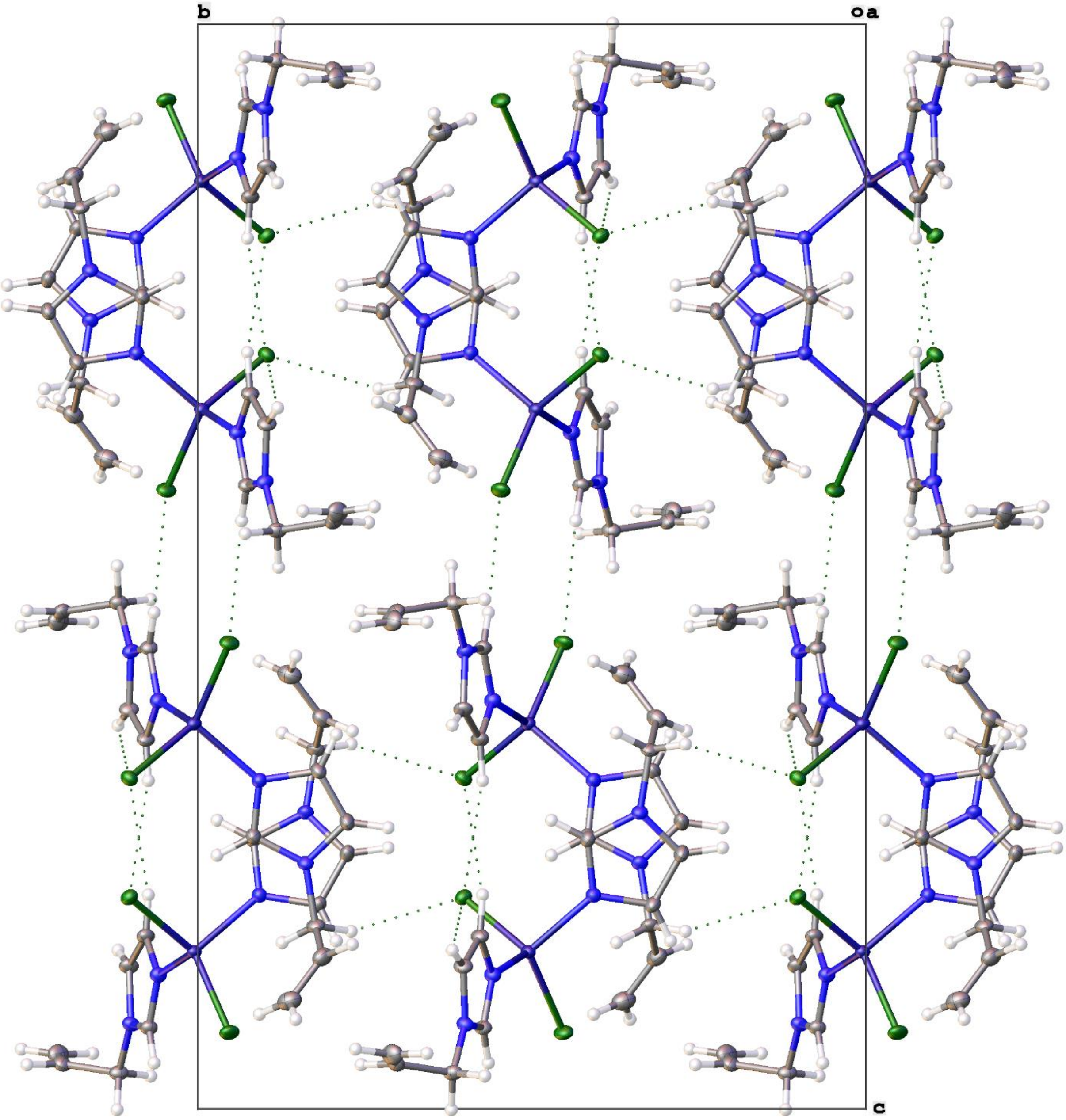

Figure S1. Crystal packing of dichlorobis(*N*-allylimidazole)zinc(II) (**1**) in a view along the a axis. ORTEP diagram was generated with displacement ellipsoids at 50% probability. H atoms are shown as spheres of arbitrary radius.

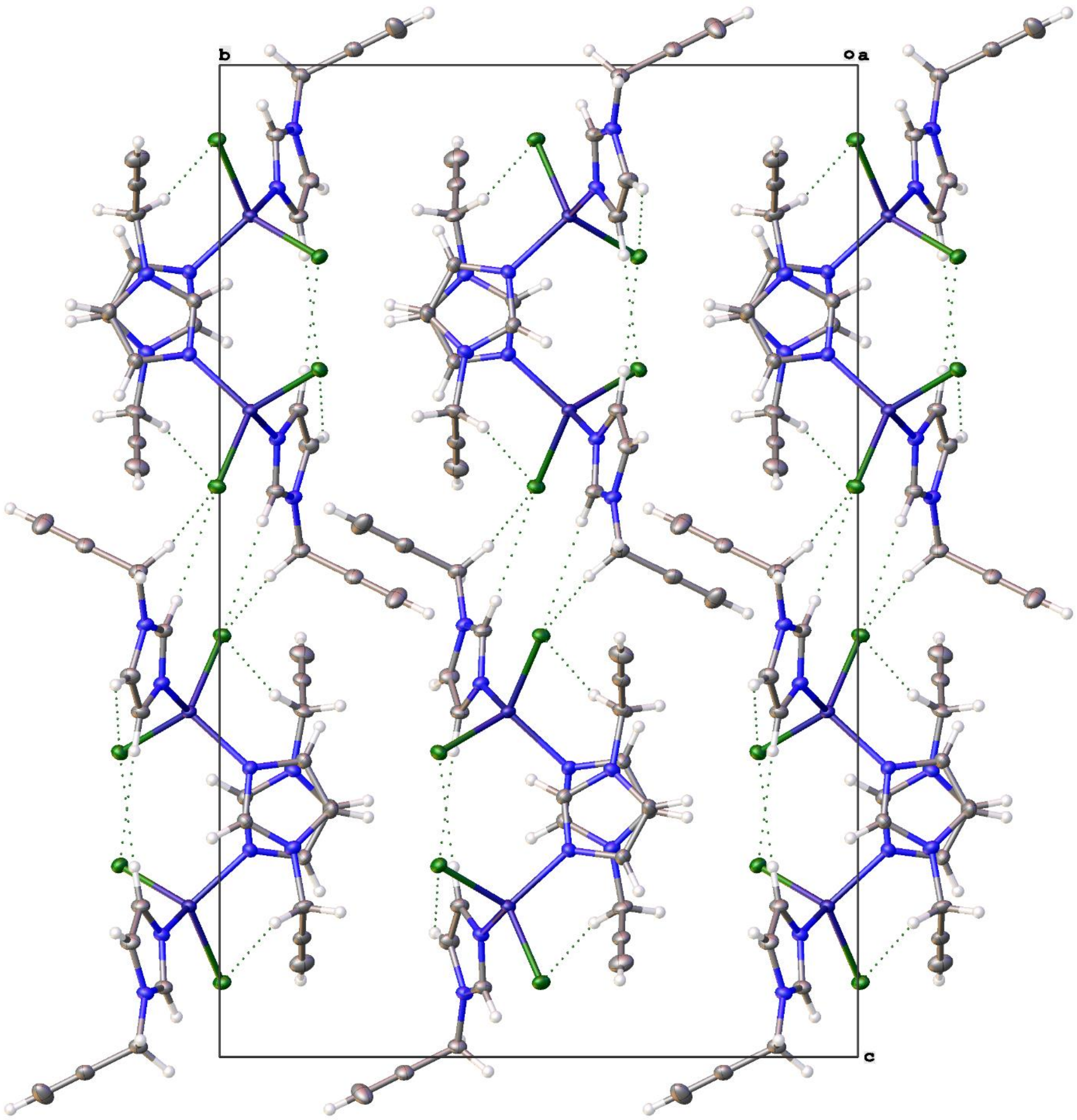

Figure S2. Crystal packing of dichlorobis(*N*-allyl-2-methylimidazole)zinc(II) (**3**) in a view along the a axis. ORTEP diagram was generated with displacement ellipsoids at 50% probability. H atoms are shown as spheres of arbitrary radius.

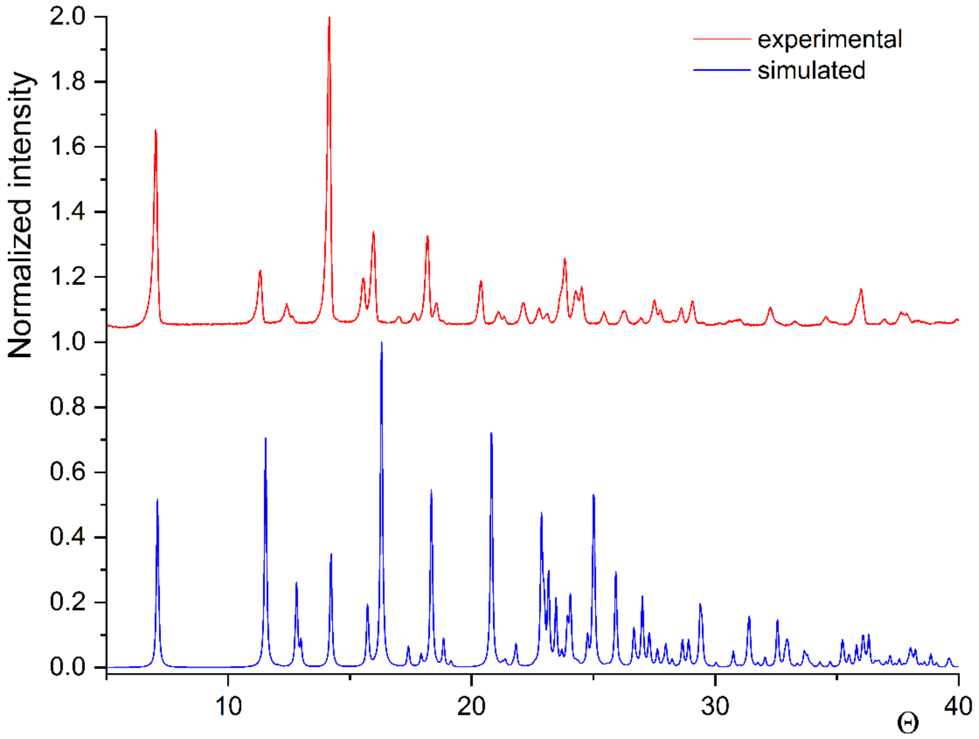

Figure S3. Experimentally obtained and simulated powder X-ray diffraction diagrams for the dichlorobis(*N*-allylimidazole)zinc(II) (**1**). The experimental curve is shifted in intensity by 1.

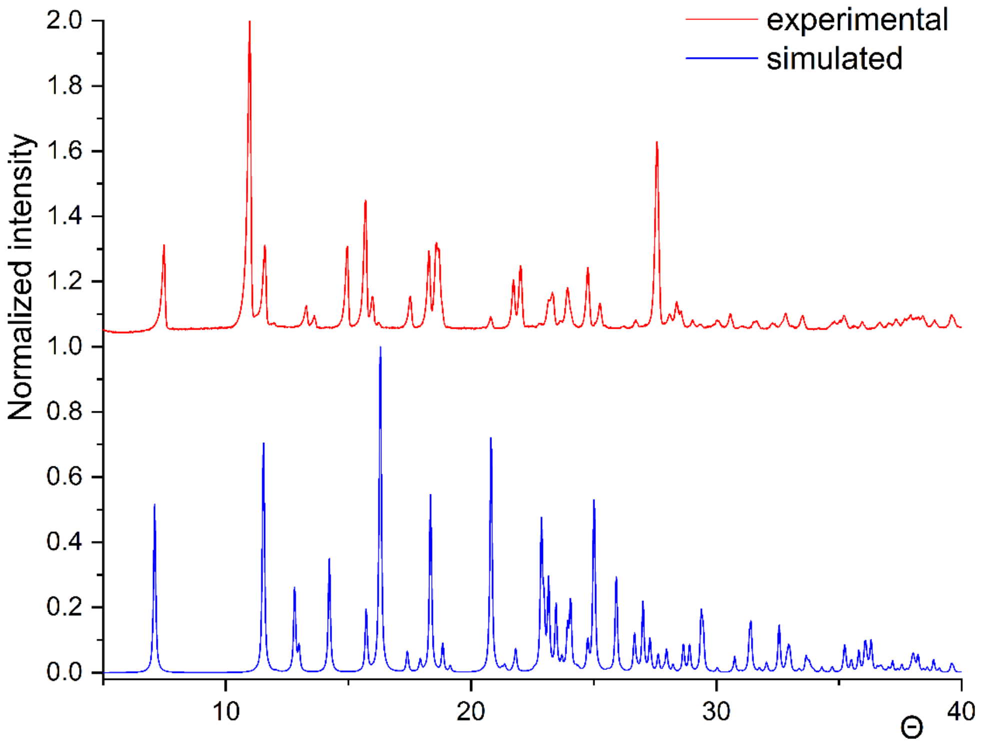

Figure S4. Experimentally obtained and simulated powder X-ray diffraction diagrams for the dichlorobis(*N*-allyl-2-methylimidazole)zinc(II) (**3**). The experimental curve is shifted in intensity by 1.

NMR spectra

Table S16. The chemical shifts of protons in NMR spectra of compounds in different solvents.

Compound	Chemical shifts								
	H2	H4	H5	H1'	H2'a	H2'b	H3'a	H3'b	CH_3
	$CDCl_3$								
N-Allylimidazole	8.02	7.01	7.21	4.62	5.97	5.97	5.4	5.32	-
N-Allyl-2-MethylImidazole		6.88	7.13	4.5	5.91	5.91	5.34	5.11	2.61
N-PropargylImidazole	8.15	7.25	7.16	4.8	-	-	2.61	-	-
N-Vinylimidazole	8.21	7.28	7.28	6.95	5.5	5.18	-	-	-
**1**	8.02	7.01	7.21	4.62	5.97	5.97	5.4	5.32	
**2**		6.88	7.13	4.5	5.91	5.91	5.34	5.11	2.61
**3**	8.15	7.25	7.16	4.8	-	-	2.61	-	-
**4**	8.21	7.28	7.28	6.95	5.5	5.18	-	-	-
	DMSO-d6								
**1**	8.08	7.06	7.36	4.74	6.02	6.02	5.25	5.16	-
**2**		6.93	7.27	4.64	4.64	5.97	5.22	4.96	2.38
**3**	8.06	7.05	7.42	5.02	-	-	3.6	-	-
**4**	8.36	6.95	7.87	7.28	5.7	5.07	-	-	-
	D_2O								
**1**	8.02	7.14	7.29	4.71	6.02	6.02	5.31	5.19	-
**2**		7.09	7.23	4.66	5.98	5.98	5.3	5.04	2.44
**3**	8.02	7.12	7.37	5.02	-	-	2.92	-	-
**4**	8.09	7.14	7.54	7.09	5.55	5.12	-	-	-

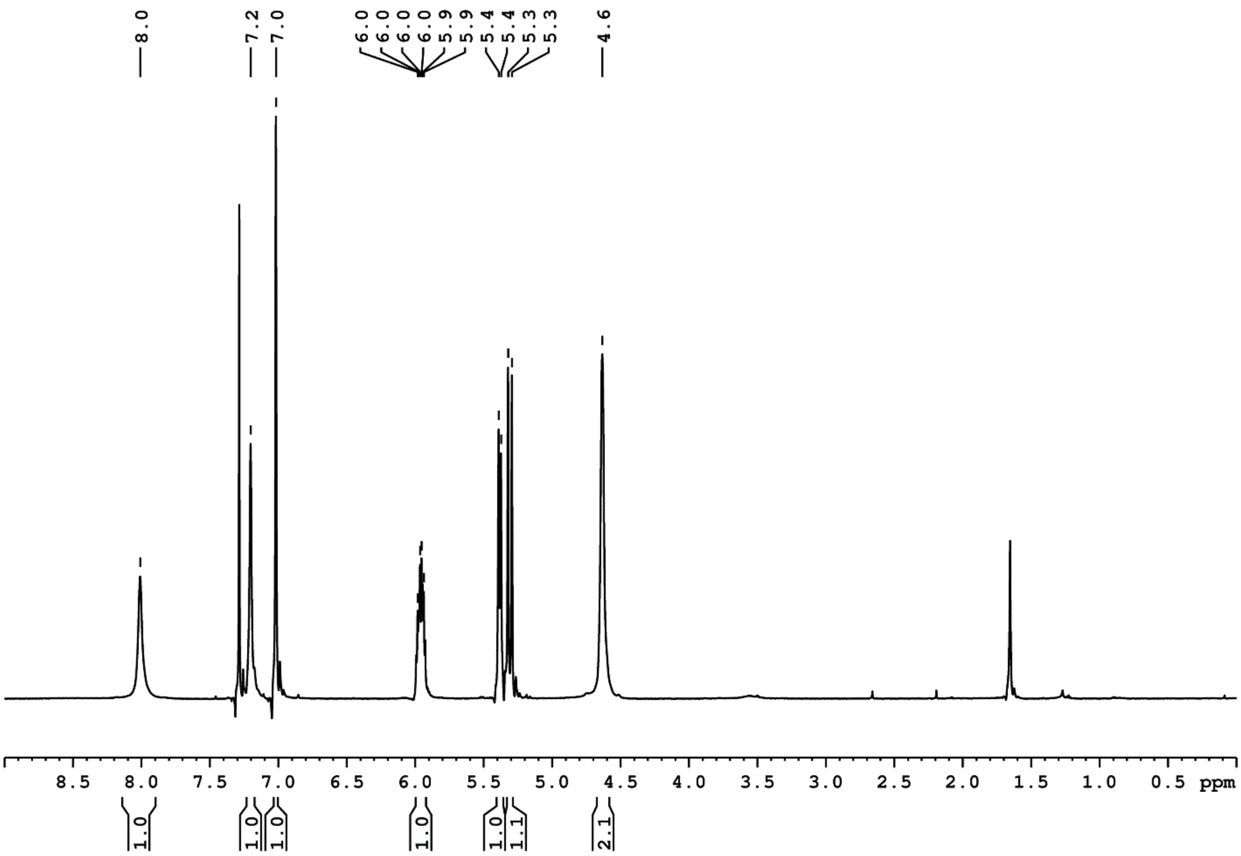

Figure S5. ^{1}H NMR spectrum of dichlorobis(*N*-allylimidazole)zinc(II) (**1**) in $CDCl_3$.

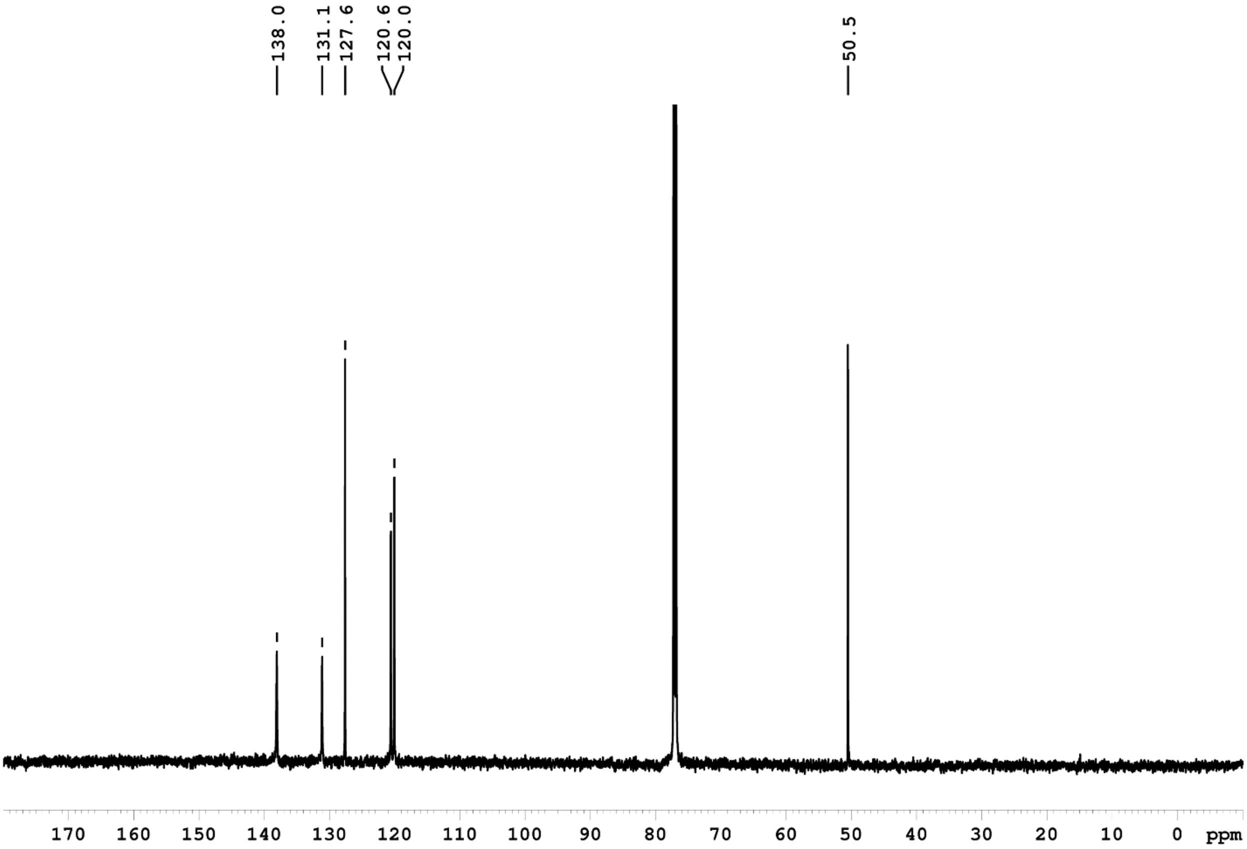

Figure S6. ^{13}C NMR spectrum of dichlorobis(*N*-allylimidazole)zinc(II) (**1**) in $CDCl_3$.

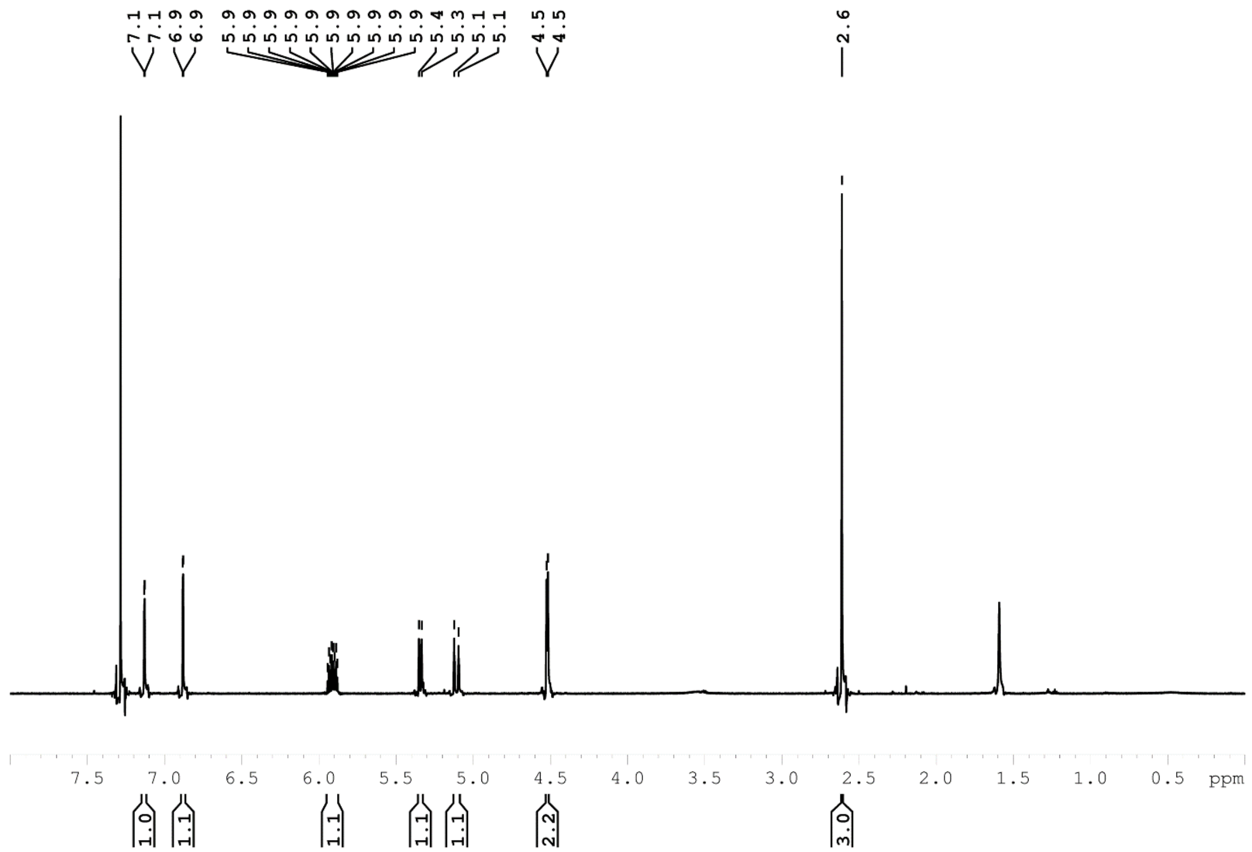

Figure S7. ^{1}H NMR spectrum of dichlorobis(*N*-allyl-2-methyl-imidazole)zinc(II) (**2**) in $CDCl_3$.

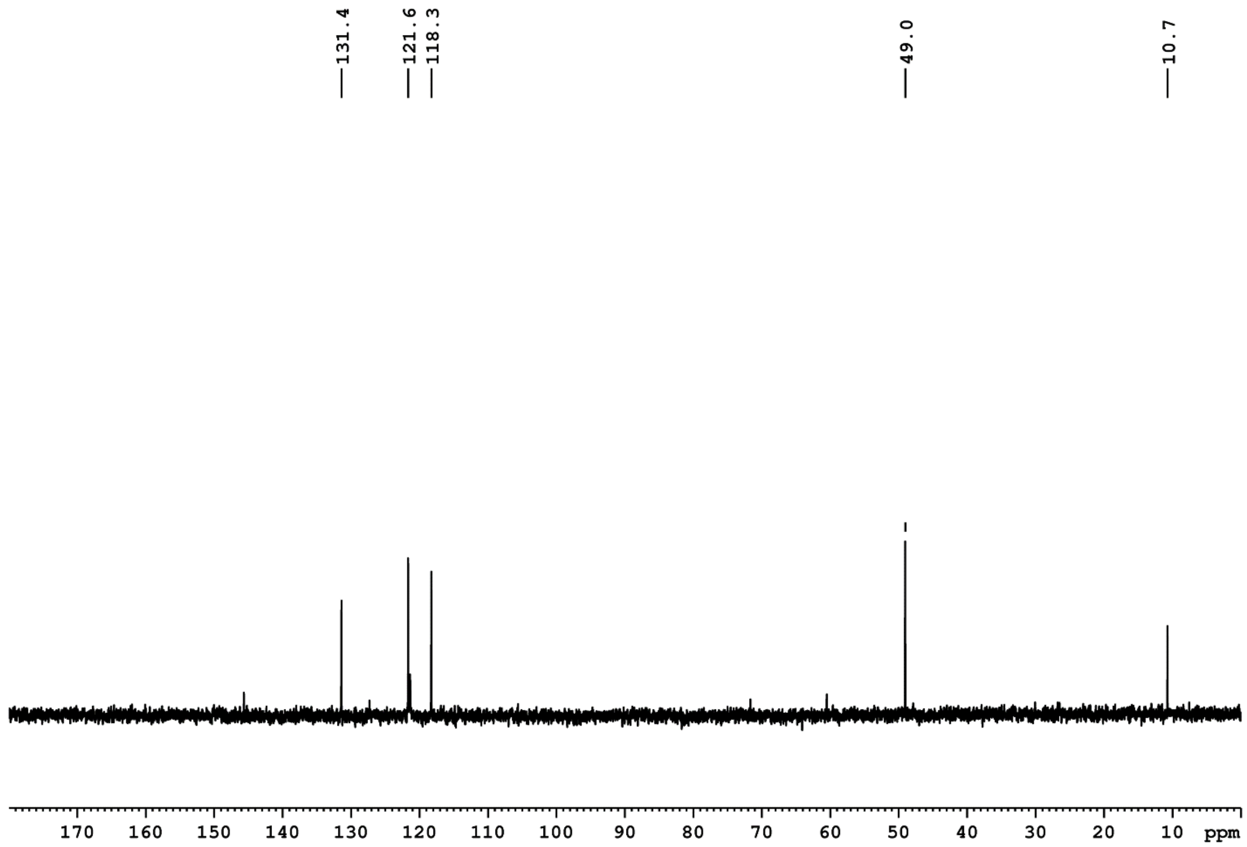

Figure S8. ^{13}C NMR spectrum of dichlorobis(*N*-allyl-2-methyl-imidazole)zinc(II) (**2**)in D_2O.

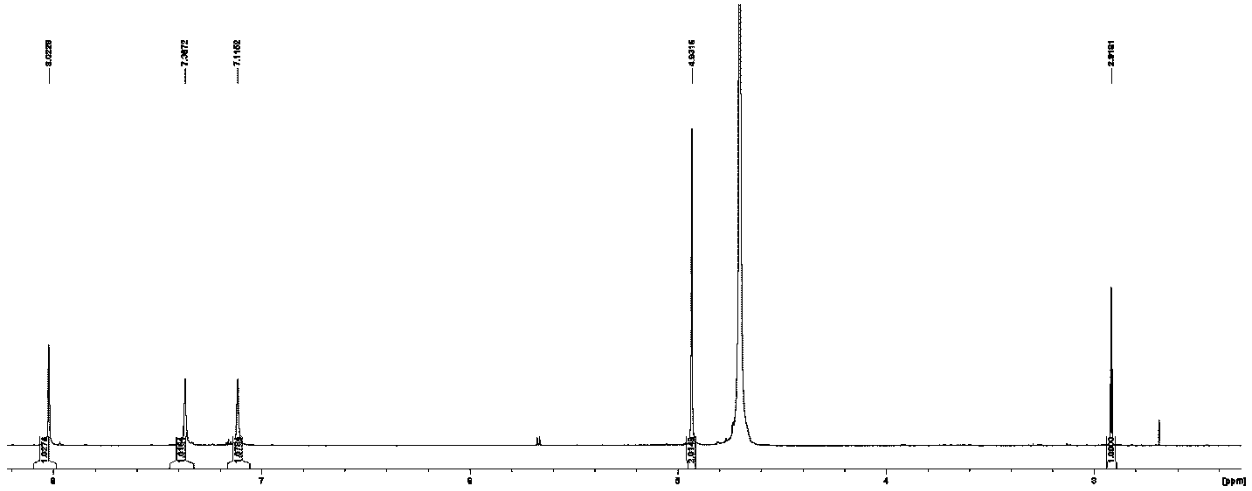

Figure S9. ^{1}H NMR spectrum of dichlorobis(*N*-allyl-2-methylimidazole)zinc(II) (**3**) in D_2O.

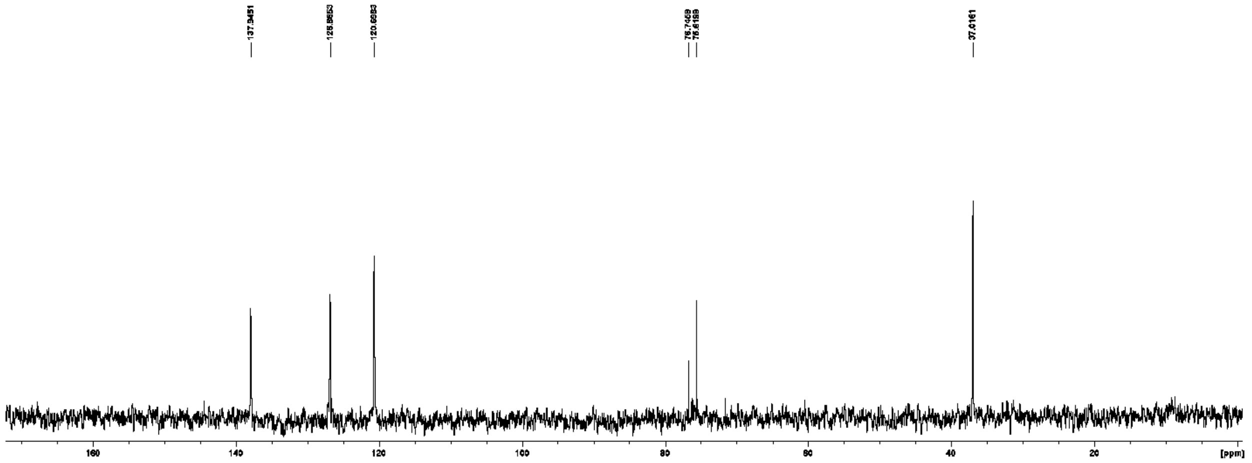

Figure S10. ^{13}C NMR spectrum of dichlorobis(*N*-allyl-2-methylimidazole)zinc(II) (**3**) in D_2O.

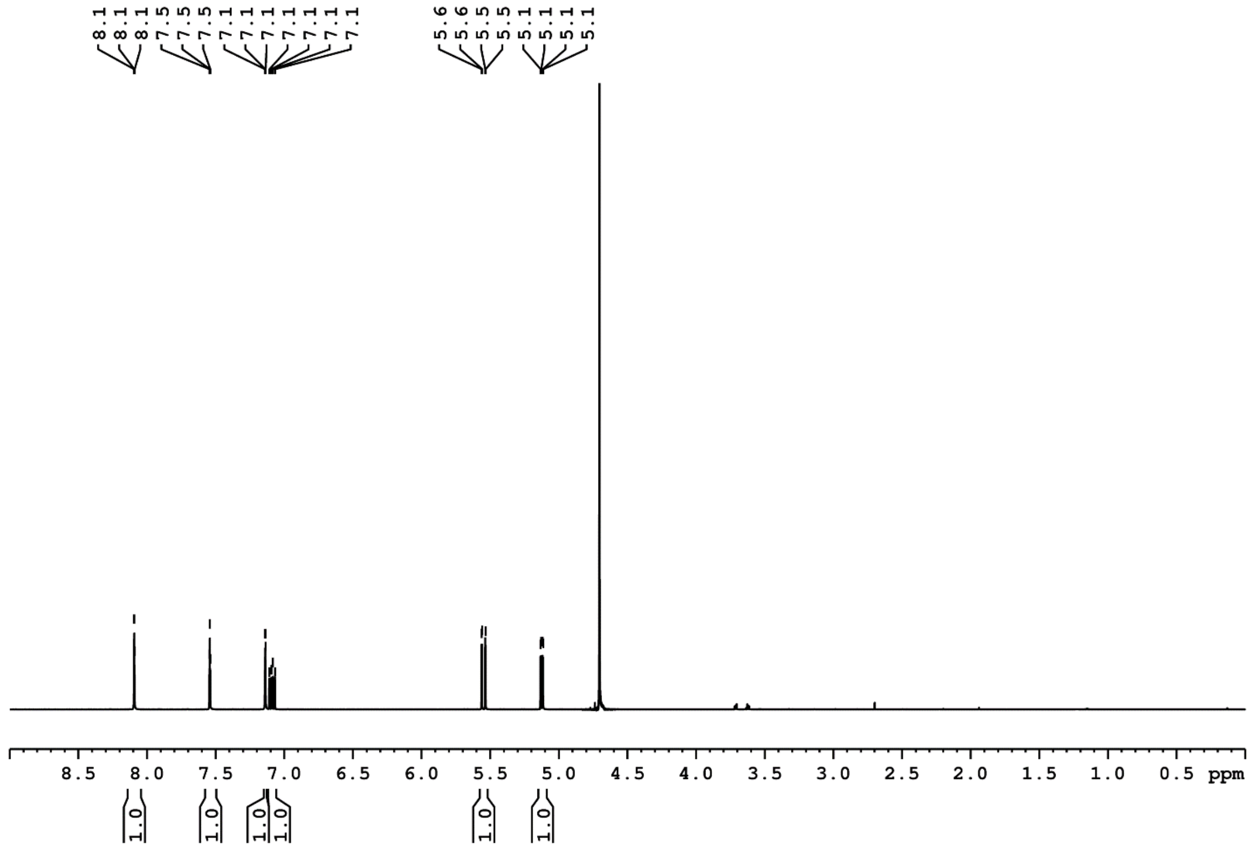

Figure S11. ^{1}H NMR spectrum of dichlorobis(*N*-vinylimidazole)zinc(II) (**4**) in D_2O.

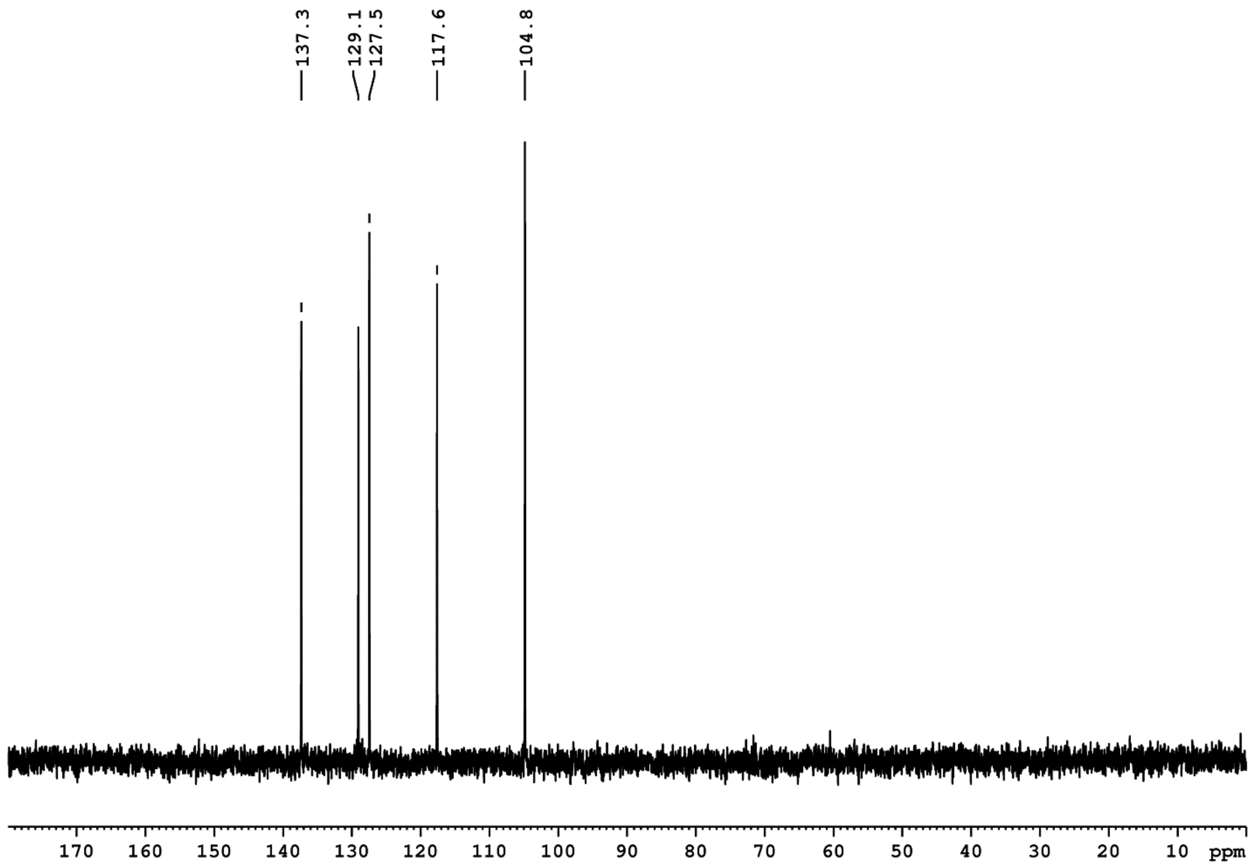

Figure S12. ^{13}C NMR spectrum of dichlorobis(*N*-vinylimidazole)zinc(II) (**4**) in D_2O.

Microphotographs

Microphotographs of planar skin wounds in rats. Staining with hematoxylin and eosin. Magnification ×200.

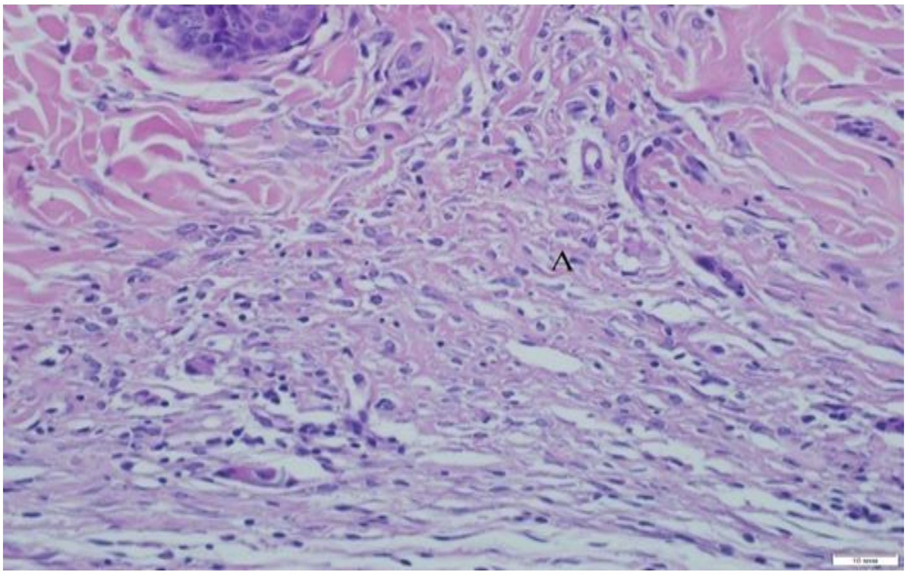

Figure S13. Control group (intact). Granulation tissue (A).

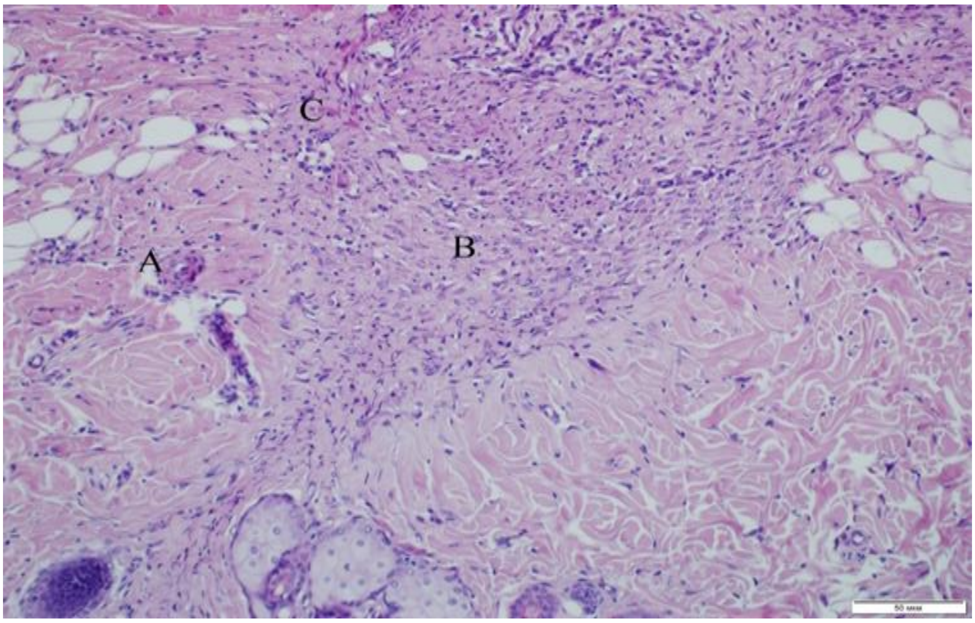

Figure S14. Exposed to Na-CMC. An arteriole with a thickened wall (A). A large amount of granulation tissue (B). Diapedesis, moderate angiogenesis (C).

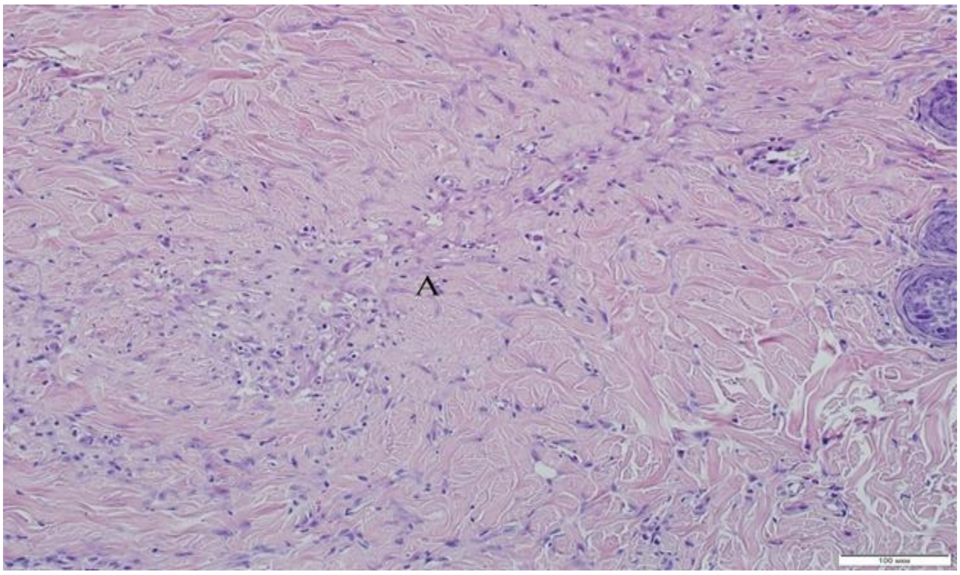

Figure S15. Exposed to $ZnSO_4$. Mature granulation tissue with a low number of cellular elements and a high content of extracellular matrix components; the tissue is present in a small amount (A)

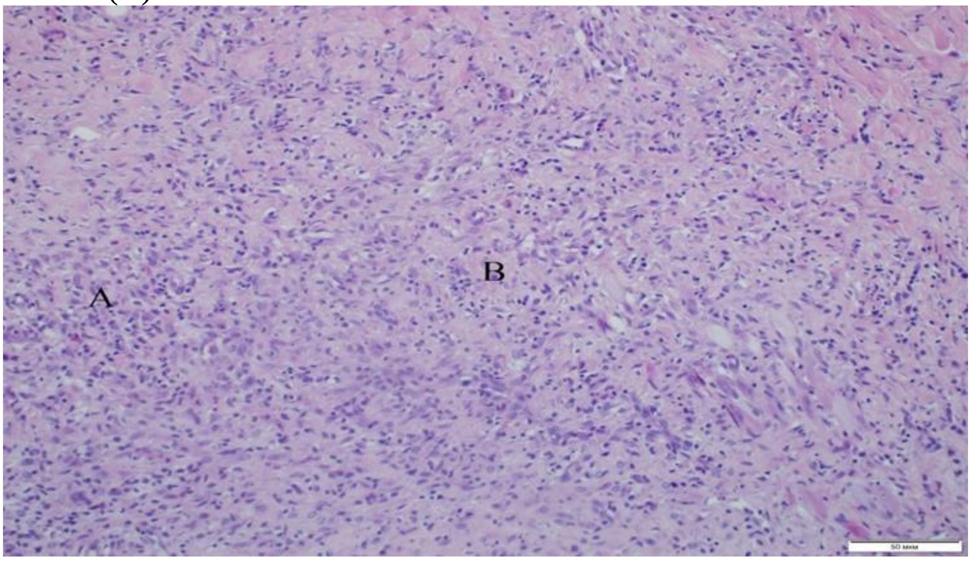

Figure S16. Exposed to **2**. Mature granulation tissue with a low number of cellular elements and a high content of extracellular matrix components; the tissue is present in a small amount (A)

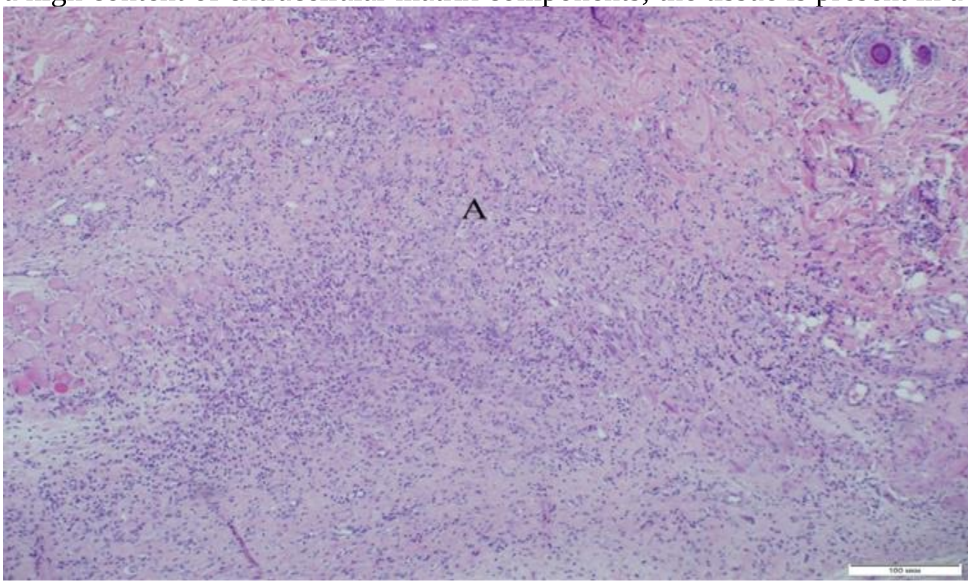

Figure S17. Exposed to **3**. Immature granulation tissue (A).

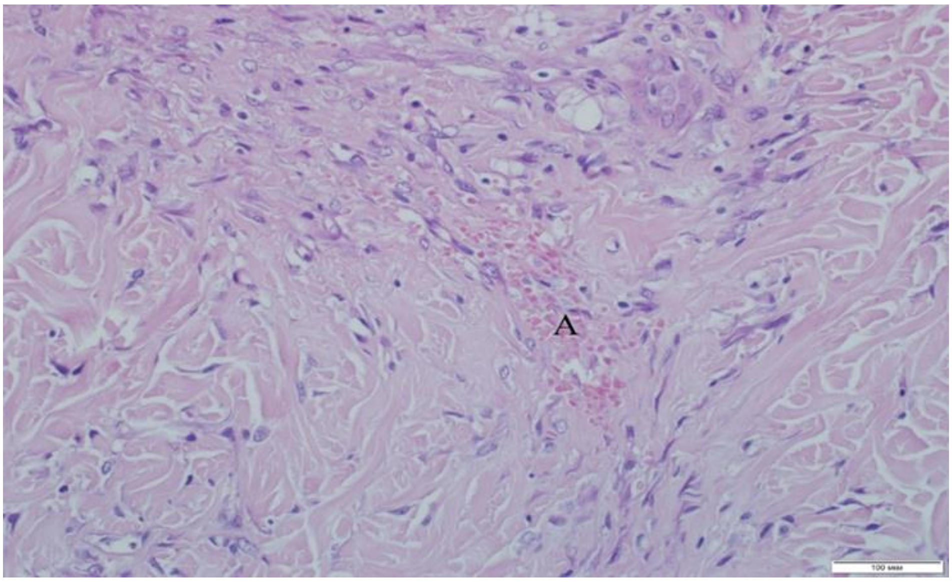

Figure S18. Exposed to **4**. Diapedesis, signs of active angiogenesis (A).

DFT calculations data

Table S17. Coordination bonds lengths, Å.

#	Complex	Calculated				Determined by X-ray			
		Zn-N1	Zn-N2	Zn-Cl1	Zn-Cl2	Zn-N1	Zn-N2	Zn-Cl1	Zn-Cl2
1	**1**	2.0277	2.0277	2.2656	2.2734	2.0020	2.0103	2.2447	2.2555
2	**2**	2.0471	2.04712	2.2595	2.2595	-	-	-	-
3	**3**	2.0331	2.0332	2.2658	2.2726	1.9941	2.0003	2.2537	2.2763
4	**4**[2]	2.0346	2.0393	2.2597	2.2614	2.0142	2.0054	2.2376	2.2518

Table S18. Basic thermochemical parameters of the complexes.

Complex	Complex formation			One ligand dissociation			One chloride dissociation		
	ΔH_f, kcal/mol	ΔG_f, kcal/mol	$\log K_f$	ΔH_{diss}, kcal/mol	ΔG_{diss}, kcal/mol	$\log K_{diss}$	ΔH_{diss}, kcal/mol	ΔG_{diss}, kcal/mol	$\log K_{diss}$
**1**	-29.02	-9.63	7.06	21.99	10.44	-7.66	9.15	2.35	-1.72
**2**	-33.8	-10.79	7.92	17.07	8.78	-6.44	10.64	1.98	-1.45
**3**	-28.36	-7.63	5.59	17	7.8	-5.72	9.54	1.84	-1.35
**4**	-26.25	-5.84	4.29	17.64	8.41	-6.17	9.61	1.77	-1.3

Table S19. Basic chemical and biological parameters of the complexes.

Complex	$\log K_f$	FMO energies			IC50, µg/mL			CC50, µg/mL	
		I	A	ΔE	*Staphylococcus aureus*	*Escherichia coli*	*Candida albicans*	Hep2c	RD
**1**	7.06	7.98	-0.17	8.15	1200	1900	300	46	24
**2**	7.92	7.70	-0.36	8.06	320	450	610	69	27
**3**	5.59	8.09	-0.25	8.34	900	1250	160	53	42
**4**	4.29	8.00	0.61	7.39	460	600	80	64	27

Table S20. Correlation coefficients between the toxicity of complexes and their electronic parameters.

Parameter	Toxicity of complexes in relation to				
	Staphylococcus aureus Wood 46	*Escherichia coli* M-17	*Candida albicans* 927	Hep2c	RD
I	0.5	0.5	0.19	-0.72	0.35
A	-0.06	-0.06	-0.31	-0.24	-0.48
ΔE	0.89	0.89	0.75	-0.86	0.21
χ	-0.89	-0.89	-0.75	0.86	-0.21
η	0.69	0.69	-0.95	0.43	0.69
σ	-0.89	-0.89	-0.75	0.86	-0.21
µ	0.89	0.89	0.75	-0.86	0.21
S	-0.89	-0.89	0.75	0.86	-0.21
ω	-0.31	-0.31	-0.97	0.45	-0.31
ΔN_{max}	-0.89	-0.89	-0.75	0.86	-0.21
p	-0.12	-0.12	0.21	0.53	-0.12

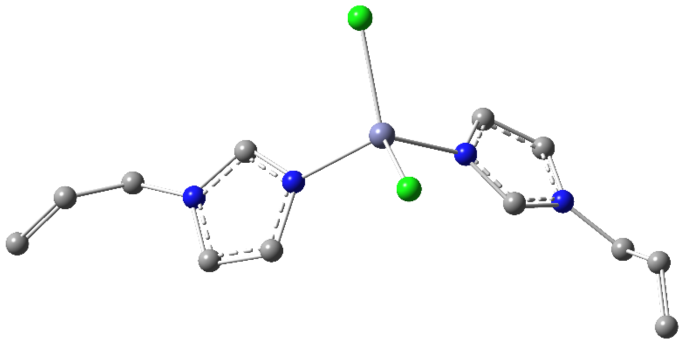

Figure S19. Geometry of **1** optimized by the DFT (MN15/6-31+G(d,p)). Hydrogen atoms are omitted for clarity.

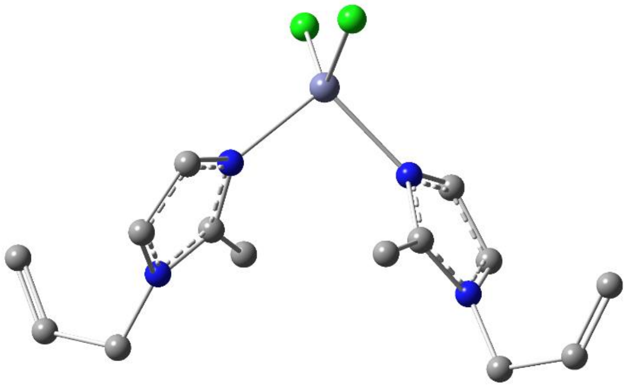

Figure S20. Geometry of **2** optimized by the DFT (MN15/6-31+G(d,p)). Hydrogen atoms are omitted for clarity.

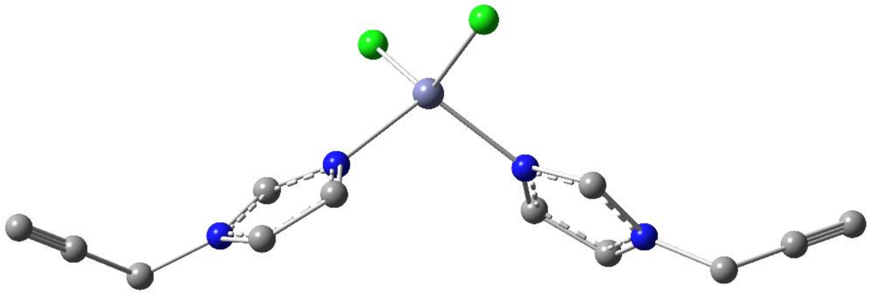

Figure S21. Geometry of **3** optimized by the DFT (MN15/6-31+G(d,p)). Hydrogen atoms are omitted for clarity.

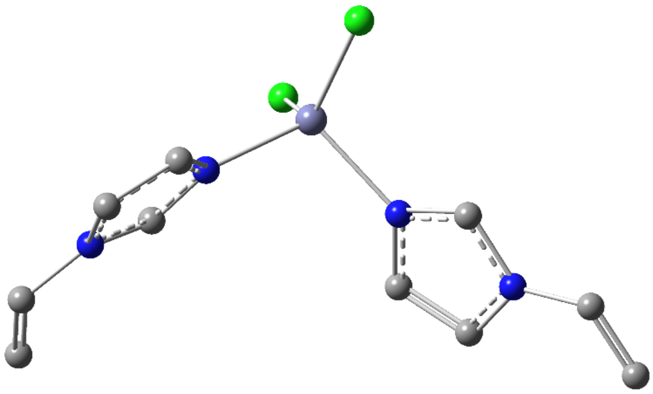

Figure S22. Geometry of **4** optimized by the DFT (MN15/6-31+G(d,p)). Hydrogen atoms are omitted for clarity.

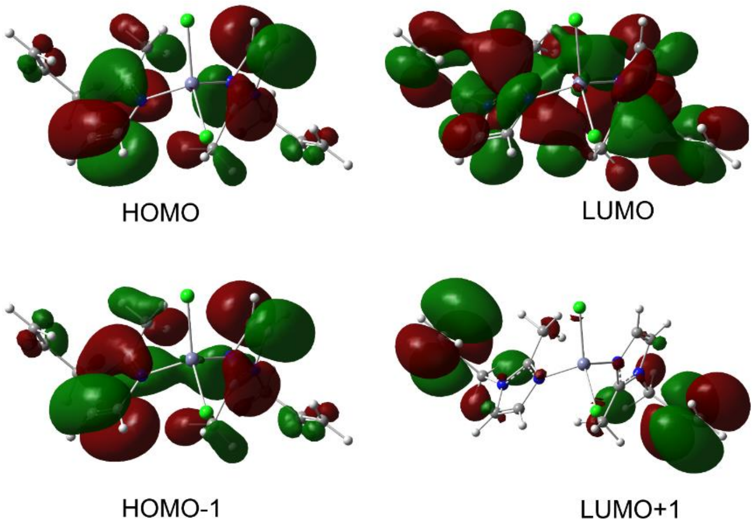

Figure S23. Frontier molecular orbitals plot for the complex **2**.

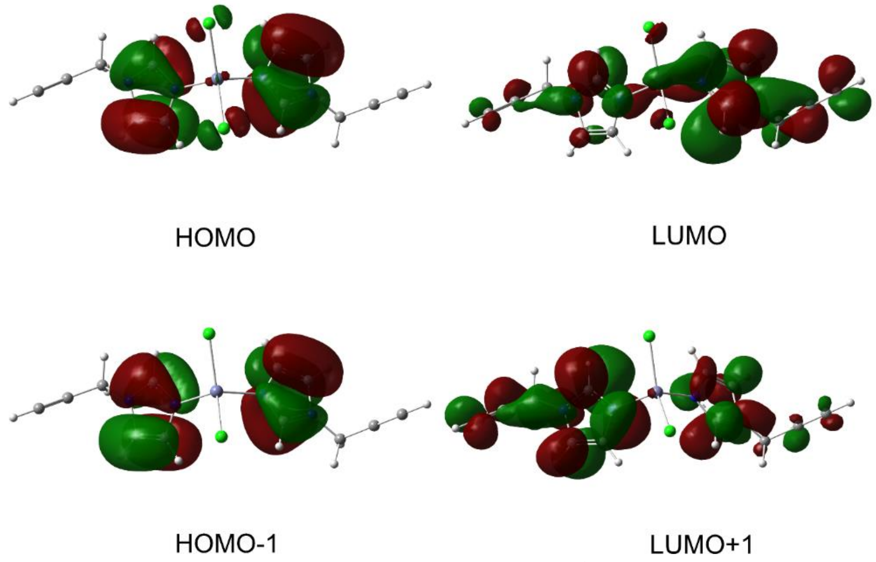

Figure S24. Frontier molecular orbitals plot for the complex **3**.

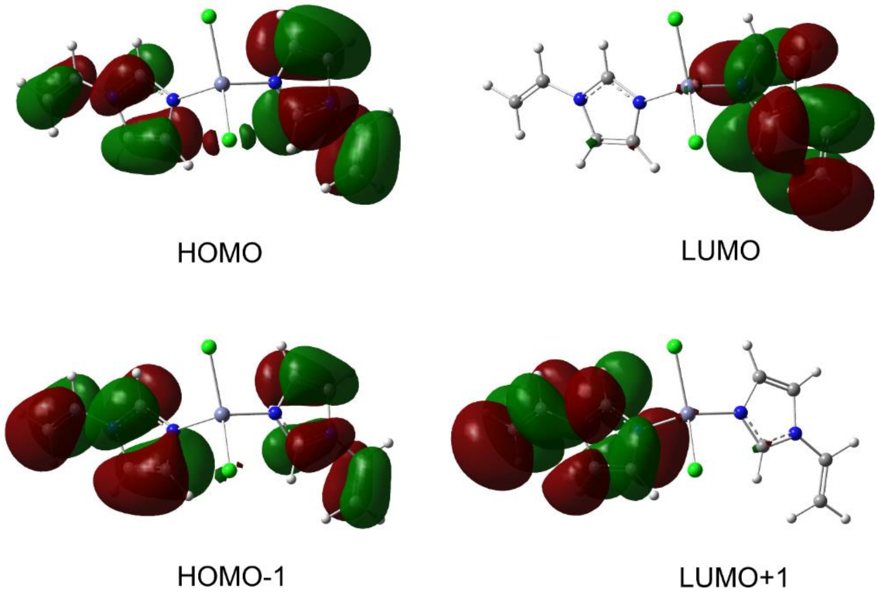

Figure S25. Frontier molecular orbitals plot for the complex **4**.

Table S21. Cartesian coordinates of dichlorobis(N-allylimidazole)zinc(II) (**1**).

Atom	x	y	z
Zn	0.144013	1.148623	0.26717
Cl	-0.41671	2.890653	-1.08168
Cl	0.859525	1.403409	2.401657
N	-3.47754	-0.85016	-0.22892
N	-1.53347	0.011227	0.329888
N	3.471281	-1.00111	-1.03267
N	1.651508	0.183993	-0.6859
C	-2.5284	0.051614	-0.54694
H	-2.58796	0.714638	-1.40007
C	-1.86012	-0.95991	1.254156
H	-1.21175	-1.18847	2.088501
C	-3.06898	-1.50276	0.917295
H	-3.66963	-2.27377	1.377512
C	-4.71832	-1.1059	-0.97027
H	-4.681	-2.11924	-1.38266
H	-4.73421	-0.39793	-1.80473
C	-5.92411	-0.9344	-0.08906
H	-6.04541	0.044959	0.373653
C	-6.81285	-1.90729	0.123655
H	-6.69506	-2.88895	-0.33233
H	-7.6859	-1.75141	0.751052
C	2.641699	-0.48186	-0.10477
H	2.783561	-0.59432	0.962088
C	1.852654	0.087556	-2.04687
H	1.183839	0.559461	-2.75297
C	2.982812	-0.64762	-2.27417
H	3.47956	-0.94334	-3.18643
C	4.652548	-1.8358	-0.76753
H	5.248116	-1.82793	-1.68556
H	4.330324	-2.86489	-0.57863
C	5.448061	-1.30536	0.391304
H	5.824845	-0.28758	0.290333
C	5.702588	-2.02281	1.48782
H	5.324833	-3.03779	1.599148
H	6.299686	-1.62167	2.301857

Table S22. Cartesian coordinates of dichlorobis(N-Allyl-2-MethylImidazole)zinc(II) (**2**).

Atom	x	y	z
Zn	-2E-06	1.77176	0.000031
N	-1.42901	0.417359	0.560537
N	-2.51696	-1.48833	0.64873
N	1.428975	0.417333	-0.56054
N	2.516971	-1.48833	-0.64885
C	-1.7565	-0.67564	-0.12573
C	-3.15945	-2.72228	0.213155
H	-3.31671	-3.3478	1.097738
H	-2.46339	-3.26744	-0.43443

C	-4.46486	-2.48219	-0.50153
H	-4.96746	-3.38819	-0.83742
C	-5.00536	-1.28397	-0.73383
H	-4.52596	-0.36148	-0.40777
H	-5.95174	-1.18943	-1.25784
C	-2.6946	-0.87178	1.873877
H	-3.28109	-1.32691	2.658642
C	-2.0061	0.305179	1.810173
C	1.756462	-0.67572	0.125639
C	2.69474	-0.87162	-1.8739
H	3.281329	-1.32663	-2.65866
C	2.006132	0.305278	-1.81016
H	1.872611	1.068934	-2.56361
C	3.159412	-2.72234	-0.21338
H	3.316778	-3.34774	-1.09803
H	2.463281	-3.26759	0.434061
C	4.464739	-2.48233	0.501489
H	4.967322	-3.38836	0.837297
C	5.005174	-1.28412	0.734031
H	4.525772	-0.36161	0.40806
H	5.951489	-1.18964	1.258174
Cl	-0.59663	2.83996	-1.89953
Cl	0.596801	2.839743	1.899662
C	1.345983	-0.99282	1.522713
H	2.18723	-1.39925	2.093552
H	0.99806	-0.07866	2.011271
H	0.528546	-1.72613	1.539348
H	-1.87256	1.068775	2.563684
C	-1.34611	-0.9926	-1.52285
H	-2.18741	-1.39889	-2.09371
H	-0.99813	-0.0784	-2.01132
H	-0.52873	-1.72597	-1.53961

Table S23. Cartesian coordinates of dichlorobis(N-PropargylImidazole)zinc(II) (**3**).

Atom	x	y	z
Zn	0.002246	1.150064	0.085099
Cl	-0.35232	2.378677	-1.78537
Cl	0.417212	1.876487	2.198104
N	-3.50315	-1.10056	-0.21909
N	-1.63486	-0.04455	0.246849
N	3.457362	-1.18354	-0.04463
N	1.638503	0.015343	-0.3257
C	-2.55696	-0.25686	-0.68075
H	-2.56873	0.180471	-1.67067
C	-2.00539	-0.78241	1.353236
H	-1.41757	-0.77287	2.260424
C	-3.16757	-1.44507	1.075798
H	-3.77916	-2.11197	1.665766
C	-4.66363	-1.58492	-0.97019

H	-4.64652	-1.10203	-1.95102
H	-4.55917	-2.66259	-1.13085
C	-5.9214	-1.29428	-0.2746
C	-6.96121	-1.0702	0.301955
H	-7.88137	-0.86755	0.810137
C	2.486098	-0.47532	0.566599
H	2.427302	-0.32692	1.637041
C	2.085267	-0.39363	-1.56609
H	1.56728	-0.11431	-2.47284
C	3.217741	-1.14088	-1.40691
H	3.867213	-1.63428	-2.11521
C	4.560287	-1.88254	0.618103
H	4.481758	-2.95279	0.403131
H	4.433048	-1.75406	1.696476
C	5.866047	-1.37118	0.188063
C	6.944396	-0.95891	-0.17408
H	7.898356	-0.59045	-0.49064

Table S24. Cartesian coordinates of dichlorobis(N-vinylimidazolyl)zinc(II) (**4**).

Atom	x	y	z
Zn	0.144013	1.148623	0.26717
Cl	-0.41671	2.890653	-1.08168
Cl	0.859525	1.403409	2.401657
N	-3.47754	-0.85016	-0.22892
N	-1.53347	0.011227	0.329888
N	3.471281	-1.00111	-1.03267
N	1.651508	0.183993	-0.6859
C	-2.5284	0.051614	-0.54694
H	-2.58796	0.714638	-1.40007
C	-1.86012	-0.95991	1.254156
H	-1.21175	-1.18847	2.088501
C	-3.06898	-1.50276	0.917295
H	-3.66963	-2.27377	1.377512
C	-4.71832	-1.1059	-0.97027
H	-4.681	-2.11924	-1.38266
H	-4.73421	-0.39793	-1.80473
C	-5.92411	-0.9344	-0.08906
H	-6.04541	0.044959	0.373653
C	-6.81285	-1.90729	0.123655
H	-6.69506	-2.88895	-0.33233
H	-7.6859	-1.75141	0.751052
C	2.641699	-0.48186	-0.10477
H	2.783561	-0.59432	0.962088
C	1.852654	0.087556	-2.04687
H	1.183839	0.559461	-2.75297
C	2.982812	-0.64762	-2.27417
H	3.47956	-0.94334	-3.18643
C	4.652548	-1.8358	-0.76753
H	5.248116	-1.82793	-1.68556

H	4.330324	-2.86489	-0.57863
C	5.448061	-1.30536	0.391304
H	5.824845	-0.28758	0.290333
C	5.702588	-2.02281	1.48782
H	5.324833	-3.03779	1.599148
H	6.299686	-1.62167	2.301857

References

1. Lin, B.; Chu, F.; Ren, Y.; Jia, B.; Yuan, N.; Shang, H.; Feng, T.; Zhu, Y.; Ding, J. Alkaline stable C2-substituted imidazolium-based cross-linked anion exchange membranes for alkaline fuel cell applications. *Journal of Power Sources* **2014**, *266*, 186-192, doi:10.1016/j.jpowsour.2014.05.003.
2. López-Saucedo, F.; Zúñiga-Villarreal, N.; Flores-Rojas, G.G.; Martínez-Otero, D.; Magariños, B.; Bucio, E. Zinc heterocyclic vinyl complexes and their gamma-irradiated derivatives: From the metal to antimicrobial materials. *Reactive and Functional Polymers* **2020**, *146*, 104410, doi:10.1016/j.reactfunctpolym.2019.104410.
3. Sterkhova, I.V.; Parshina, L.N.; Grishchenko, L.A.; Borodina, T.y.N.; Belovezhets, L.A.; Semenov, V.A. Complexes of zinc(II) chloride and acetate with propargylimidazoles: synthesis, structure and non-covalent interactions. *Structural Chemistry* **2023**, *34*, 2249-2262, doi:10.1007/s11224-023-02245-6.
4. Krause, L.; Herbst-Irmer, R.; Sheldrick, G.M.; Stalke, D. Comparison of silver and molybdenum microfocus X-ray sources for single-crystal structure determination. *Journal of Applied Crystallography* **2015**, *48*, 3-10, doi:doi:10.1107/S1600576714022985.
5. Sheldrick, G. SHELXT - Integrated space-group and crystal-structure determination. *Acta Crystallographica Section A* **2015**, *71*, 3-8, doi:doi:10.1107/S2053273314026370.
6. Sheldrick, G. Crystal structure refinement with SHELXL. *Acta Crystallographica Section C* **2015**, *71*, 3-8, doi:10.1107/S2053229614024218.
